# Merkel Cell Polyomavirus Small T Antigen Induces Cancer and Embryonic Merkel Cell Proliferation in a Transgenic Mouse Model

**DOI:** 10.1371/journal.pone.0142329

**Published:** 2015-11-06

**Authors:** Masahiro Shuda, Anna Guastafierro, Xuehui Geng, Yoko Shuda, Stephen M. Ostrowski, Stefan Lukianov, Frank J. Jenkins, Kord Honda, Stephen M. Maricich, Patrick S. Moore, Yuan Chang

**Affiliations:** 1 Cancer Virology Program, University of Pittsburgh Cancer Institute, University of Pittsburgh, Pittsburgh, Pennsylvania, United States of America; 2 Richard King Mellon Institute for Pediatric Research, Department of Pediatrics, University of Pittsburgh, Pittsburgh, Pennsylvania, United States of America; 3 Department of Pathology, University of Pittsburgh School of Medicine, Pittsburgh, Pennsylvania, United States of America; 4 Department of Dermatology, Case Western Reserve University School of Medicine, Cleveland, Ohio, United States of America; Rutgers University, UNITED STATES

## Abstract

Merkel cell polyomavirus (MCV) causes the majority of human Merkel cell carcinomas (MCC) and encodes a small T (sT) antigen that transforms immortalized rodent fibroblasts *in vitro*. To develop a mouse model for MCV sT-induced carcinogenesis, we generated transgenic mice with a flox-stop-flox MCV sT sequence homologously recombined at the *ROSA* locus (*ROSA* ^*sT*^), allowing Cre-mediated, conditional MCV sT expression. Standard tamoxifen (TMX) administration to adult *Ubc* ^*CreERT2*^; *ROSA* ^*sT*^ mice, in which *Cre* is ubiquitously expressed, resulted in MCV sT expression in multiple organs that was uniformly lethal within 5 days. Conversely, most adult *Ubc* ^*CreERT2*^; *ROSA* ^*sT*^ mice survived low-dose tamoxifen administration but developed ear lobe dermal hyperkeratosis and hypergranulosis. Simultaneous MCV sT expression and conditional homozygous p53 deletion generated multi-focal, poorly-differentiated, highly anaplastic tumors in the spleens and livers of mice after 60 days of TMX treatment. Mouse embryonic fibroblasts from these mice induced to express MCV sT exhibited anchorage-independent cell growth. To examine Merkel cell pathology, MCV sT expression was also induced during mid-embryogenesis in Merkel cells of *Atoh1* ^*CreERT2/+*^; *ROSA* ^*sT*^ mice, which lead to significantly increased Merkel cell numbers in touch domes at late embryonic ages that normalized postnatally. Tamoxifen administration to adult *Atoh1* ^*CreERT2/+*^; *ROSA* ^*sT*^ and *Atoh1* ^*CreERT2/+*^; *ROSA* ^*sT*^; *p53f* ^*lox/flox*^ mice had no effects on Merkel cell numbers and did not induce tumor formation. Taken together, these results show that MCV sT stimulates progenitor Merkel cell proliferation in embryonic mice and is a bona fide viral oncoprotein that induces full cancer cell transformation in the *p53*-null setting.

## Introduction

Merkel cell polyomavirus (MCV) is clonally integrated into ~80% of Merkel cell carcinomas (MCC) and is currently the only human polyomavirus known to be oncogenic [1, 2]. This virus was the first human pathogen discovered by a nondirected RNAseq method called digital transcriptome subtraction [3], an approach that is now commonly used to discover and characterize human viruses [4]. The MCV T antigen locus encodes two major overlapping transcripts for the large T (LT) and small T (sT) antigen oncoproteins [5, 6]. All MCV-MCC tumors examined to date not only carry clonally-integrated virus but also possess clonal tumor-specific LT mutations that truncate the C-terminus and disrupt the helicase functions of the LT protein [5, 7, 8]. p53 targeting through a bi-partite interaction domain of the LT antigen of the closely related Simian vacuolating 40 (SV40) virus is essential for SV40 LT’s transformation activity. However, no analogous p53 targeting domain has been identified for either MCV LT or sT oncoproteins [9–11].

MCV sT is a promiscuous E3 ligase inhibitor [12, 13] and alone is sufficient to transform rodent fibroblast cell lines [14]. MCV sT rodent cell transformation does not require active protein phosphatase 2A (PP2A)-binding activity [14]. Instead, a region called the large T stabilization domain (LSD) binds and inhibits diverse E3 ligase recognition proteins including Fbw7, cdc20 and cdh1 [12, 13]. The latter two proteins are recognition subunits for the anaphase promoting complex/cyclosome (APC/C) and sT expression promotes mitotic prometaphase arrest [13]. This in turn increases CDK1-directed phosphorylation of 4E-BP1 and activation of mitosis-associated cap-dependent translation (MACT) [14]. Mutations to the MCV sT LSD eliminate MCV sT-induced transformation, and knockdown of either LT or sT initiates cell death or cell cycle arrest of MCV-positive MCC cells [15, 16].

Recently, Spurgeon et al demonstrated that transgenic, keratin 14 promoter-driven MCC-derived genomic T antigen expression in mouse skin induces papillomatosis [17]. Similarly, Verhaegen et al demonstrated that transgenic MCV sT expression in mice using a keratin 5 (K5) promoter induces hyperproliferative lesions that mimic human squamous cell carcinoma *in situ* [18]. This hyperplasia is dependent on an intact MCV sT LSD region. To date, however, no mouse models have demonstrated that transgenic MCV T antigen expression induces full neoplasia.

We generated transgenic mice that conditionally express MCV sT from the *ROSA26* locus to measure the oncogenic potential of this viral protein. We confirm that MCV sT expression induces a hyperplastic response in skin tissues as previously described. We further demonstrate that only prolonged MCV sT expression in a p53-null context produces highly anaplastic, poorly differentiated malignancies in internal organs. This requirement for multiple oncogenic contributions for full transformation is similar to that seen for c-Myc, Wnt-1 and SV40 LT [19–21]. We also found that MCV sT induction in Merkel cells of embryonic mice led to transient increases in Merkel cell numbers but was insufficient to cause proliferation or tumorigenesis in adult Merkel cell populations regardless of p53 status.

## Results

### Generation of MCV sT Transgenic Mouse

**Fig 1:**
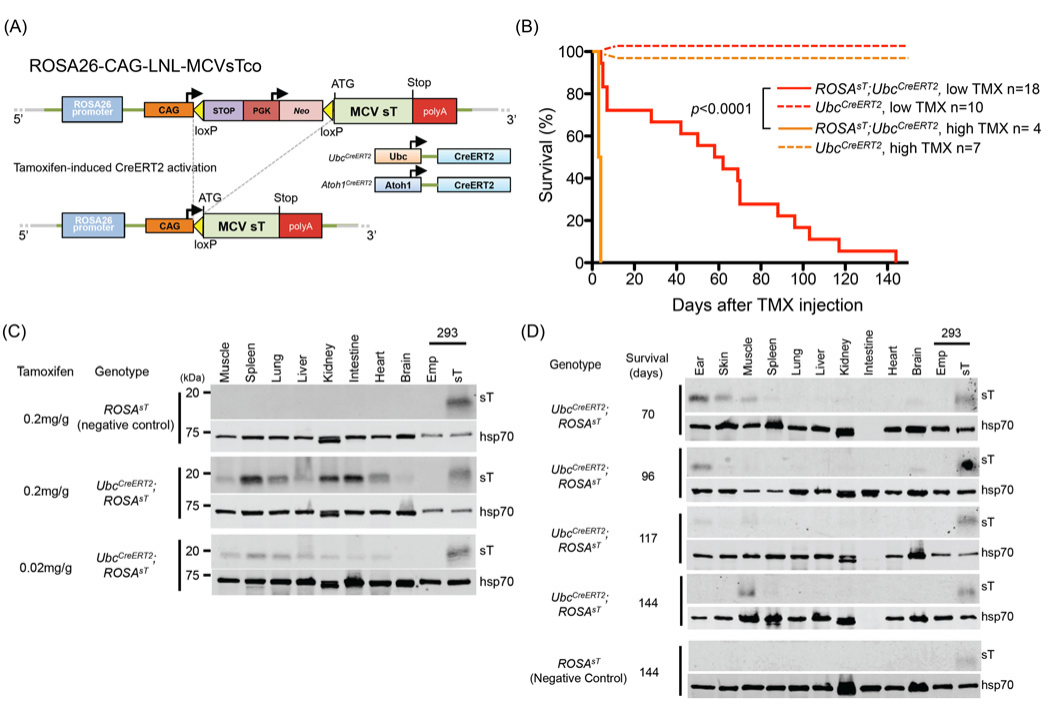
MCV sT expression is lethal in mice. (A) Design of the *ROSA26-CAG-LNL-MCVsTco*, a ROSA26 knock-in construct encoding codon-optimized MCV sT with flanking loxP-STOP-Neo-loxP (LNL) sequence, recombined with the ROSA26 genomic locus. *ROSA26-CAG-LNL-MCVsTco* crossed with *Ubc* ^*CreERT2*^ or *Atoh1* ^*CreERT2*^ mice allows for TMX-induced Cre recombinase excision of the loxP-STOP-Neo-loxP sequence that results in MCV sT expression under the CAG promoter. (B) MCV sT expression is lethal in *Ubc* ^*CreERT2*^ *; ROSA* ^*sT*^ transgenic mice. Kaplan-Meier curve of high dose (0.2 mg/g) and low dose (0.02 mg/g) TMX injected mice. High dose TMX injection caused rapid weight loss and mice reached the euthanasia criterion (20% loss of body weight) within 5 days of the injection (n = 4, solid orange). Survival of the control *Ubc* ^*CreERT2*^ mice (n = 7, dotted orange) was not affected by TMX injection. *Ubc* ^*CreERT2*^ *; ROSA* ^*sT*^ mice injected with lower dose TMX exhibited significantly prolonged survival compared to high dose TMX (n = 18, solid red) and control mice were unaffected (n = 10, dotted red) (C) Multi-tissue MCV sT protein expression in *Ubc* ^*CreERT2*^ *; ROSA* ^*sT*^ mice that died immediately after high dose TMX injection (< 5 days). Lower dose TMX injection induces less MCV sT protein expression in a mouse that died at day 7 after the injection as detected by immunoblotting using an antibody raised against MCV sT, CM8E6 [22]. MCV sT vector or empty vector transfected HEK293 cells were used as a positive and a negative control, respectively. Equal amounts of sT-transfected HEK293 cell lysates were loaded for normalization across different blots. Hsp70/Hsc70 was detected as a protein quality control. (D) MCV sT protein expression was maintained in tissues of *Ubc* ^*CreERT2*^ *; ROSA* ^*sT*^ mice that survived over 70 days after low dose TMX injection. Immunoblots were performed as in (C). Tissue lysates from *ROSA* ^*sT*^ were used as a negative control.

A transgenic mouse model with inducible MCV sT expression, *ROSA* ^*sT*^, was generated in the C57BL/6 genetic background. To conditionally express MCV sT protein, codon-optimized MCV sT (sTco) cDNA was vector cloned downstream of a *loxP-*flanked neomycin phosphotransferase cDNA sequence with a 5’ sequence homologous to the mouse *ROSA26* locus to generate *ROSA26-CAG-LNL-MCVsTco* (Fig 1A). *ROSA26-CAG-LNL-MCVsTco* was delivered by homologous recombination into the ROSA26 locus of mouse embryonic stem (ES) cells (see details in materials and methods).

### High Level Expression of MCV sT in Tissues Is Lethal to Mice

To conditionally induce cre-loxP recombination and sT expression in multiple organs, *ROSA* ^*sT*^ mice were mated to *Ubc* ^*CreERT2*^ mice encoding human ubiquitin C promoter-driven Cre recombinase fused to a triple mutant form of the human estrogen receptor activatable by tamoxifen (TMX). We examined sT expression at two different TMX dosing levels: high-dose TMX activation to promote wide-spread sT expression, and low-dose TMX activation in which a stochastic fraction of cells in most tissues would undergo recombination and sT expression.

High-dose CreERT2 activation by a single intraperitoneal (i.p.) TMX injection (0.2 mg per gram of mouse body weight) to adult *Ubc* ^*CreERT2*^; *ROSA* ^*sT*^ mice induced rapid weight loss in all mice tested (n = 4). These mice became dehydrated, less active on day 3 after injection and reached the 20% weight loss euthanasia endpoint within 5 days. None of the control mice negative for the *Ubc* ^*CreERT2*^ transgene showed appreciable weight loss after TMX injection (Fig 1B). *Ubc* ^*CreERT2*^ *; ROSA* ^*sT*^ mice did not show weight loss in the absence of TMX injection, and their survival was comparable to *Ubc* ^*CreERT2*^ and *ROSA* ^*sT*^ control mice.

**Fig 2:**
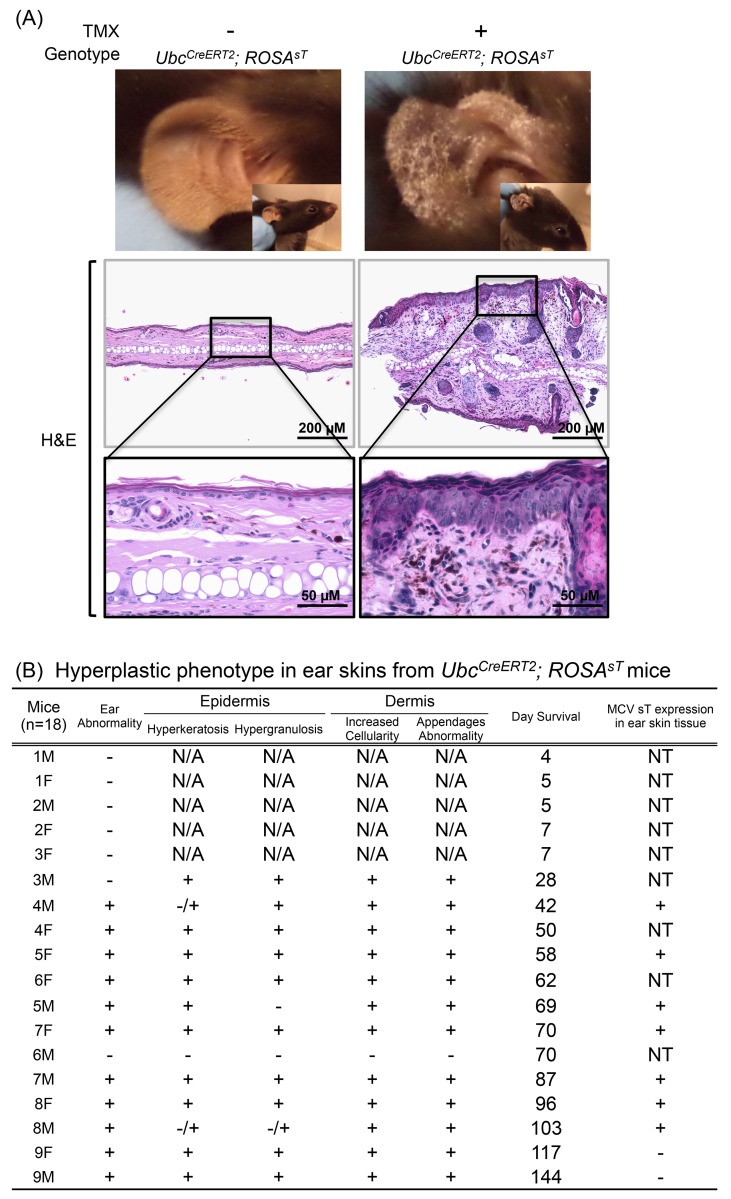
MCV sT induces hyperproliferaton of acral skin. (A) Hyperplastic phenotype in ear skin from *Ubc* ^*CreERT2*^ *; ROSA* ^*sT*^ mice. Ear skin tissues from *Ubc* ^*CreERT2*^ *; ROSA* ^*sT*^ mice were subjected to H&E staining. (B) Hyperproliferative phenotype in ear skin of *Ubc* ^*CreERT2*^ *; ROSA* ^*sT*^ mice. H&E stained ear skin sections from low-dose TMX injected *Ubc* ^*CreERT2*^ *; ROSA* ^*sT*^ and *ROSA* ^*sT*^ mice were randomized and evaluated in a blinded manner by two pathologists for hyperkeratosis/hypergranulosis in the epidermis and increased cellularity/appendage abnormalities. N/A: not available since ear tissues were normal and not harvested. NT: not tested.

Low-dose TMX, at 10% of the high dose (0.02 mg/g), markedly reduced lethality, with 72% (13/18) of mice surviving 10 or more days (n = 18) (Figs 1B and 2B) despite a steady weight loss during the course of the experiment. One such mouse survived 144 days post TMX injection before reaching the 20% weight loss euthanasia criterion and this was then considered the endpoint for the study period.

Regardless of the TMX dose, tissue immunoblotting of *Ubc* ^*CreERT2*^; *ROSA* ^*sT*^ mice revealed widespread MCV sT expression in muscle, spleen, lung, liver, kidney, intestine, heart and brain tissues of mice that died within 10 days after TMX injection whereas low dose TMX induced less sT protein tissue expression (Fig 1C and S1A Fig). No sT expression was detected in *ROSA* ^*sT*^ littermate control mice. For mice injected with low-dose TMX and surviving >10 days, however, MCV sT protein expression in various tissues was reduced compared to those surviving <10 days, with sT protein detected only in ear, skin, muscle and brain tissues by immunoblotting (Fig 1D). MCV sT expression was not detectable except in muscle for *Ubc* ^*CreERT2*^; *ROSA* ^*sT*^ mice sacrificed on day 144 post low-dose TMX injection. Increased numbers of TUNEL positive epithelial cells were detected in the intestine from *Ubc* ^*CreERT2*^; *ROSA* ^*sT*^ mice receiving multiple high-dose TMX (once a day for 3 days), as compared to *ROSA* ^*sT*^ control mice (S1 Fig). Other organs including spleen, lung and kidney showed similar results (data not shown).

### Low Dose MCV sT Expression Induces Dermal and Epidermal Hyperplasia in Ear Tissues

Of the *Ubc* ^*CreERT2*^; *ROSA* ^*sT*^ mice that survived over 42 days post TMX injection (n = 12), gross external examination showed striking bilateral and diffuse thickening of auricles. MCV sT protein was detected by immunoblotting of ear tissues in 77.8% of mice (7/9, three ear tissues were not harvested), and sT induction was associated with hyperproliferation of the dermis and epidermis by microscopy in 61.1% (11/18) of *Ubc* ^*CreERT2*^; *ROSA* ^*sT*^ mice (Fig 2A). MCV sT protein expression levels, however, were not associated with this phenotype. Two mice surviving for over 117 days (9F and 9M) exhibited a hyperplastic ear phenotype despite lack of detectable sT protein expression in the ears. Neither *ROSA* ^*sT*^ nor *Ubc* ^*CreERT2*^ control mice demonstrated this ear phenotype. Hematoxylin & eosin (H&E) staining of thickened ear tissue showed epithelial hyperkeratosis (90.9%, 10/11) and hypergranulosis (81.8%, 9/11) (Fig 2). Other than gross and microscopic ear abnormalities, we detected no other hyperproliferative lesion in this group of sT-expressing mice.

### MCV sT Expression Induces Increased Proliferation in Mouse Embryonic Fibroblasts

**Fig 3:**
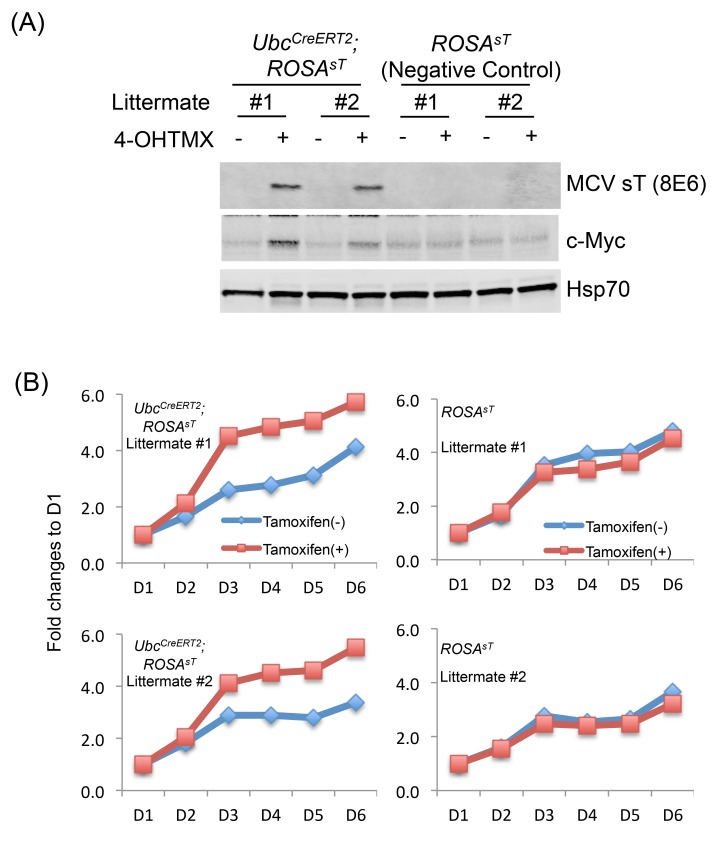
MCV sT induces hyperproliferaton in MEF cells. (A) Induction of MCV sT in multiple mouse embryonic fibroblast (MEF) cells from littermate by 4-OHTMX. MEFs extracted from *Ubc* ^*CreERT2*^ *; ROSA* ^*sT*^ and *ROSA* ^*sT*^ embryos were cultured in the presence or absence of 500 nM 4-OHTMX for 7 days. MCV sT and c-Myc protein expression was detected by immunoblot. Hsp/Hsc70 protein expression was detected as a loading control. (B) MCV sT expression accelerates cell proliferation in MEF cells. Proliferation of MEF cells treated with or without 4-OHTMX was evaluated by Wst1 assays.

To investigate the dermal fibroblastic proliferation and to determine whether MCV sT affects cell proliferation in this mouse model, we isolated mouse embryonic fibroblasts (MEFs) from *Ubc* ^*CreERT2*^; *ROSA* ^*sT*^ and *ROSA* ^*sT*^ embryos. *In vitro* treatment with 500 nM 4-hydroxy tamoxifen (4-OHTMX) induced MCV sT expression in *Ubc* ^*CreERT2*^; *ROSA* ^*sT*^ MEFs (n = 8 embryos) but not *ROSA* ^*sT*^ MEFs (n = 2 embryos) (Fig 3A). Some MEFs showed leaky MCV sT expression even in the absence of 4-OHTMX treatment (S2A Fig). c-Myc expression, which has been shown to be stabilized by MCV sT [12], was also increased by MCV sT expression (Fig 3 and S2B Fig). Wst-1 cell proliferation assay demonstrated that accelerated growth occurred in *Ubc* ^*CreERT2*^; *ROSA* ^*sT*^ but not *ROSA* ^*sT*^ MEFs (Fig 3 and S2B Fig).

### MCV sT Expression in p53 Null Mice Produces Anaplastic, Poorly-Differentiated Neoplasia Expressing Mixed Lineage Markers in Spleen and Liver

To circumvent the lethality produced by MCV sT expression as reflected by high TUNEL indices in tissues and rapid death from weight loss of experimental mice, we crossed *Ubc* ^*CreERT2*^; *ROSA* ^*sT*^ mice with *p53* ^*flox/flox*^ mice to generate *Ubc* ^*CreERT2*^; *ROSA* ^*sT*^; *p53* ^*flox/flox*^ mice. In these mice, TMX administration simultaneously induced MCV sT expression and *p53* gene deletion. Nevertheless, when high dose (0.2 mg/g) TMX was delivered i.p., MCV sT expression was detected in multiple tissues and all mice died within 5 days (n = 9), suggesting that *p53* deletion does not rescue lethality induced by high levels of sT expression (S3A Fig). As with p53 wild-type mice, short-term survival rates for *Ubc* ^*CreERT2*^; *ROSA* ^*sT*^; *p53* ^*flox/flox*^ mice were greater for low-dose compared to high-dose TMX treated mice (S3A Fig) and 42.3% (11/26) mice died within 10 days of low dose TMX injection. Widespread MCV sT protein expression was also detected in multiple tissues from these mice that died within 10 days of low dose TMX injection (S3B Fig). *Ubc* ^*CreERT2*^; *ROSA* ^*sT*^; *p53* ^*flox/flox*^ mice also exhibited the hyperkeratosis/hypergranulosis ear skin phenotype seen in p53 wild-type, sT-expressing mice (S4 Fig).

**Fig 4:**
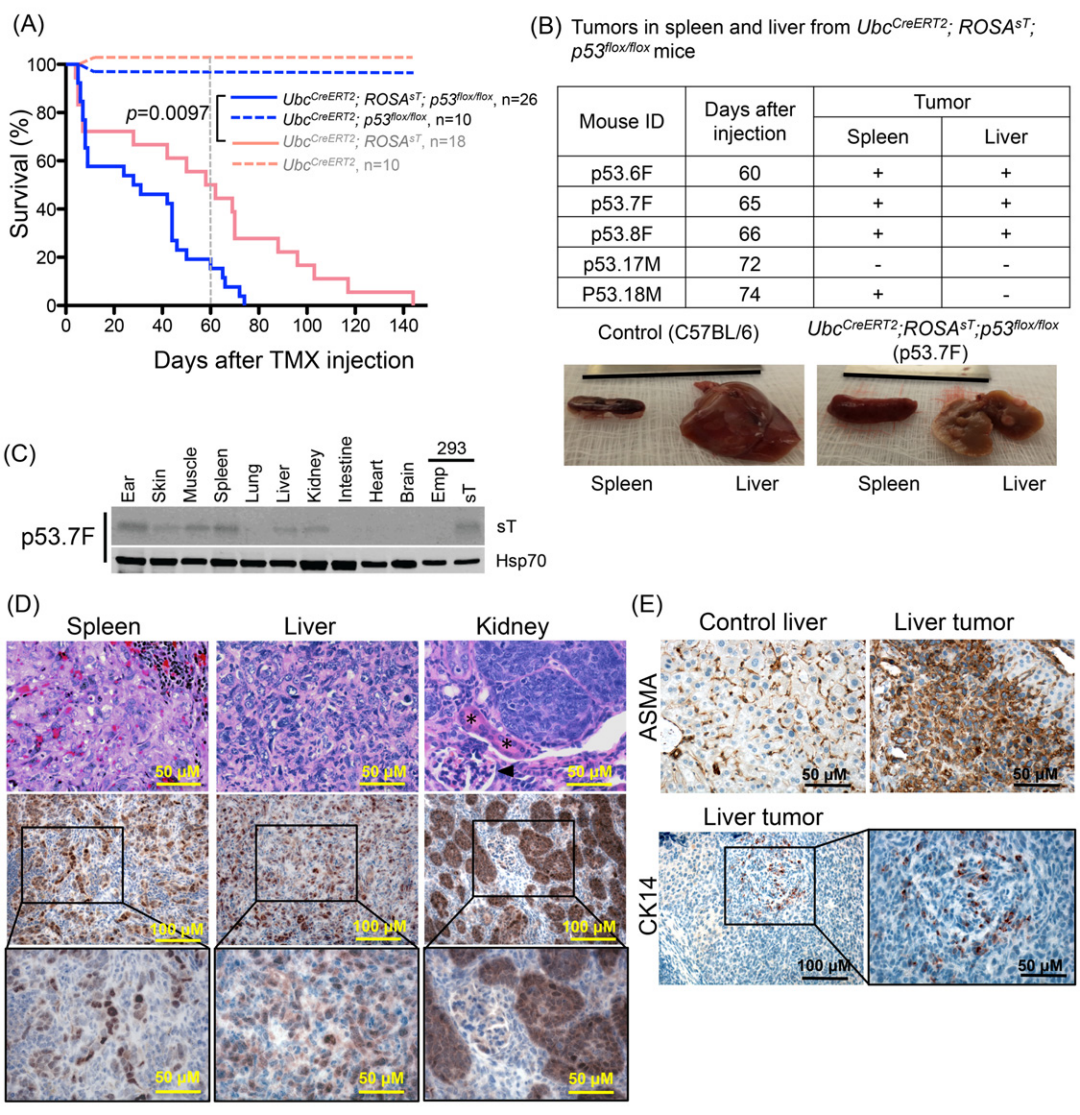
MCV sT transgenic mice develop tumors in a p53 null setting. (A) p53 ablation does not rescue mice from MCV sT-induced lethality. Kaplan-Meier curve of low dose (0.02 mg/g) TMX injected *Ubc* ^*CreERT2*^ *; ROSA* ^*sT*^ *; p53* ^*flox/flox*^ mice (Solid blue line, n = 26) and *Ubc* ^*CreERT2*^ *; p53* ^*flox/flox*^ mice (dotted blue line, n = 10). Pink lines indicate the survival of *Ubc* ^*CreERT2*^ *; ROSA* ^*sT*^ *(solid line)* and *Ubc* ^*CreERT2*^ (dotted line) mice with wild type p53 as shown in Fig 1B for comparison with p53 knockout background (blue lines). (B) MCV sT expression produces tumors *in vivo* in *p53* null setting. 80% (4/5) of mice that survived over 60 days after TMX injection develop grossly visible tumor in the spleen and liver. Representative spleen and liver tissues with tumor nodules from p53.7F are shown with corresponding normal tissues from a C57BL/6 control mouse. (C) Immunoblotting of MCV sT protein expression in liver and spleen tissues from mouse p53.7F) with macroscopic tumors. Spleen, muscle and ear tissues consistently maintained sT expression over 60 days after TMX injection. sT expression was detectable in liver tissues by immunoblotting only from liver with visible nodules (mouse p53.7F). MCV sT protein was detected using CM8E6 antibody, and Hsp/Hsc70 expression was used as a loading control. (D) The top panel shows anaplastic neoplasia in the spleen and liver. A range of proliferative changes was observed in the kidney, where distal tubular epithelia are most severely affected, but glomeruli (black arrowhead), proximal tubular epithelia (*), and interstitial tissues are relatively spared of proliferative changes. The middle panel shows a representative immunohistochemical staining of sT protein expression in liver tumor, spleen tumor and kidney tissues from *Ubc* ^*CreERT2*^ *; ROSA* ^*sT*^ *; p53* ^*flox/flox*^ mice. Tissue samples were immunostained with MCV sT (CM5E1) antibody. (E) The top panel shows a sT-induced liver tumor with immunoreactivity to α-smooth muscle actin (ASMA) and bottom panel shows K14 positivity in scattered tumor cells.

Approximately 19% of *Ubc* ^*CreERT2*^; *ROSA* ^*sT*^; *p53* ^*flox/flox*^ mice (5/26) survived 60 or more days post-low-dose TMX treatment (Fig 4A). Four of these five (80%) mice developed high-grade tumors in internal organs visible at necropsy; three mice had tumors in both the spleen and liver, and one had grossly detectable tumor nodules in the spleen alone (Fig 4B). By light microscopy, the tumor cells had highly pleomorphic nuclei that varied dramatically in size and shape with multiple nucleoli and hyperchromatism. Mitoses were numerous and the cells had indistinct cytoplasmic boundaries. An immunoblotting survey of tissues (Fig 4C and S5 Fig) demonstrated that sT was strongly expressed in these tumors whereas sT expression was not generally detectable in spleens and livers from long-lived *Ubc* ^*CreERT2*^; *ROSA* ^*sT*^ mice or the tumor-negative *Ubc* ^*CreERT2*^; *ROSA* ^*sT*^; *p53* ^*flox/flox*^ mouse (Fig 1D and S5 Fig). sT protein expression in splenic tumors was detectable in 75% (3/4) of tumor bearing mice (S5 Fig). sT expression was also detected by immunoblotting of a representative liver nodule taken from an affected mouse (p53.7F, Fig 4C). For some of these mice (2/5), MCV sT was also expressed in kidney tissues and histopathologic examination showed marked proliferative changes in tubular epithelia ranging from *in situ* lesions to fully malignant lesions with invasion through basement membranes. Despite microscopic evidence for renal neoplasia, discrete kidney tumors were not detectable by gross examination at necropsy. Immunohistochemistry showed strong expression of MCV sT localizing to the malignant lesions from the liver and spleen, and in hyperproliferative and malignant epithelial lesions of the kidney (Fig 4D). These results were reproduced at 1/2 low dose TMX (0.01 mg/g) injection. Mouse survival was inversely related to TMX dosing at 0.01, 0.02, and 0.2 mg/g (S3 Fig). 87.5% (7/8) of *Ubc* ^*CreERT2*^ *; ROSA* ^*sT*^ *; p53* ^*flox/flox*^ mice that survived over 60 days with 0.01 mg/g TMX also developed high grade tumors in spleen and/or liver tissues (data not shown). We did not observe tumorigenesis by p53 gene deletion alone in *Ubc* ^*CreERT2*^ *; p53* ^*flox/flox*^ mice up to 140 days after 0.02 mg/g TMX treatment (Fig 4A). While germ line deletion of p53 in this mouse model promotes tumor formation as early as 50 days, it has been shown that postnatal p53 gene deletion leads to a tumor incidence of only 3% even 600 days after Cre recombinase transduction, indicating that the timing of p53 loss is a critical determinant for tumor incidence [23, 24].

To determine the histogenesis of sT-induced neoplasia, tumors from *Ubc* ^*CreERT2*^; *ROSA* ^*sT*^; *p53* ^*flox/flox*^ mice were immunostained with markers for various lineages: CD45 (pan-lymphocyte), CD68 (macrophage), α-smooth muscle actin (ASMA-smooth muscle), cytokeratin 14 (epithelial), cytokeratin 8 (epithelial), neural adhesion molecule (NCAM). Tumor cells were uniformly ASMA positive. Scattered tumor cells showed positivity to CK14 and NCAM (Fig 4E). We saw no staining with the other markers including CD68 and CD45 (S6 Fig).

### p53 Ablation Is Required for Full Transformation of MEFs by MCV sT

**Fig 5:**
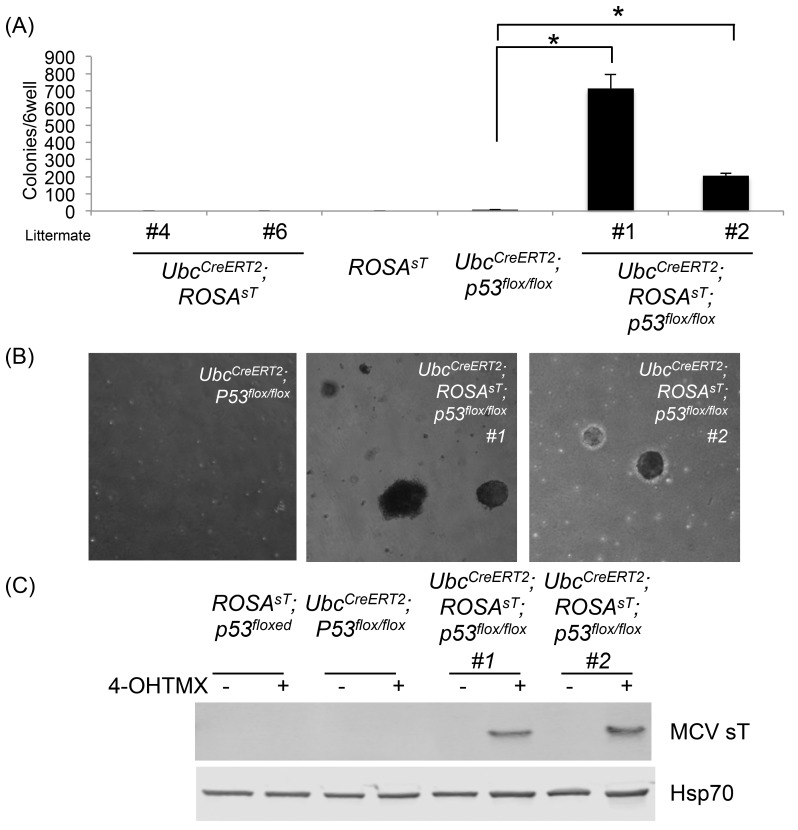
p53 ablation is critical for MCV sT transformation of MEF cells. (A) MCV sT induces soft agar colony formation in the absence of p53 in MEF cells. MEF cells isolated form *Ubc* ^*CreERT2*^ *; ROSA* ^*sT*^, *ROSA* ^*sT*^, *Ubc* ^*CreERT2*^ *; p53* ^*flox/flox*^ and two *Ubc* ^*CreERT2*^ *; ROSA* ^*sT*^ *; p53* ^*flox/flox*^ embryos from littermate were treated with 500nM 4OHTMX over 7 days, and cells were subjected to soft agar colony formation assay. Asterisks(*) indicate statistical significance *p<0*.*05*. (B) Soft agar colonies induced by p53 ablation. p53 ablation alone did not lead to colony formation. (C) Expression of MCV sT in MEFs from *Ubc* ^*CreERT2*^ *; ROSA* ^*sT*^ *; p53* ^*flox/flox*^.

**Fig 6:**
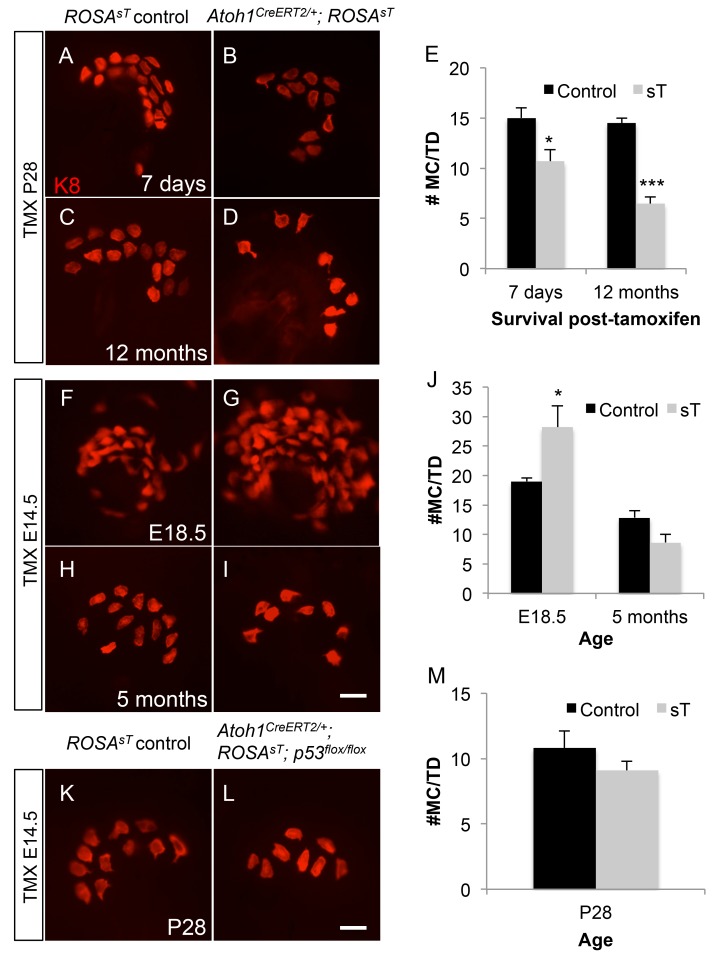
MCV sT expression increases Merkel cell numbers only during embryogenesis. Wholemount, K8-immunostained skin showing touch domes from control littermate (A, C, F, H, K), *Atoh1* ^*CreERT2/+*^; *ROSA* ^*sT*^ (B, D, G, I) and *Atoh1* ^*CreERT2/+*^; *ROSA* ^*sT*^; *p53* ^*flox/flox*^ (L) mice given TMX at P28 (A-D) or E14.5 (F-I, K, L). Age at tissue harvest is shown for each pair of panels. (E, J, M) Graphs showing Merkel cell counts per touch dome for accompanying panels. *p < 0.05, ***p<0.001. Scale bar: 10μm.

Given that MCV sT is a transforming oncoprotein in p53-null mice, we performed transformation assays using isolated MEFs from *Ubc* ^*CreERT2*^; *ROSA* ^*sT*^, *Ubc* ^*CreERT2*^; *ROSA* ^*sT*^; *p53* ^*flox/flox*^, *Ubc* ^*CreERT2*^; *p53* ^*flox/flox*^ and *ROSA* ^*sT*^ mice. Upon 4-OHTMX treatment, clones of MEFs isolated from two independent *Ubc* ^*CreERT2*^; *ROSA* ^*sT*^; *p53* ^*flox/flox*^ mice formed colonies 4 weeks after single cell seeding in soft agar culture (Fig 5A–5C). Neither MCV sT expression alone (*Ubc* ^*CreERT2*^; *ROSA* ^*sT*^) nor p53 knockout alone (*Ubc* ^*CreERT2*^; *p53* ^*flox/flox*^) induced colony formation (Fig 6B). These results are consistent with our *in vivo* tumorigenesis results.

### MCV sT Expression Increases Embryonic Merkel Cell Precursor Proliferation but Does Not Affect Adult Merkel Cell Numbers or Cause Tumorigenesis

MCC expresses phenotypic markers most similar to those expressed by normal, resident skin Merkel cells. To examine the effect of sT in this specific cell type, we targeted sT to Merkel cells by breeding *ROSA* ^*sT*^ mice to *Atoh1* ^*CreERT2/+*^ mice [25]. Atoh1 is an Atonal group proneural basic helix-loop-helix transcription factor with anti-oncogenic function [26] that plays a critical role in Merkel cell development [27]. The *Atoh1* ^*CreERT2/+*^; *ROSA* ^*sT*^ mice and control *ROSA* ^*sT*^ littermates were given a single dose of tamoxifen (0.25 mg/g) via oral gavage at postnatal day 28 (P28) to activate sT expression specifically in Merkel cells [28]. Back skin was harvested 7 days or 12 months later and immunostained for the Merkel cell marker keratin 8 (K8). At both time points, the number of Merkel cells per touch dome was lower in *Atoh1* ^*CreERT2/+*^; *ROSA* ^*sT*^ mice compared to *Atoh1* ^*+/+*^; *ROSA* ^*sT*^ control mice, consistent with previously reported effects of heterozygosity at the *Atoh1* locus [28] (Fig 6A–6E). At no time did we find touch domes with exceptionally high Merkel cell numbers, ectopic Merkel cells or evidence of tumor formation. These data suggest that sT expression alone in adult Merkel cells does not induce cell division or carcinogenesis.

Mitotic *Atoh1*+ progenitor cells produce mature Merkel cells during late embryogenesis [28]. To study the effects of sT expression on this population, we administered tamoxifen to pregnant dams at E14.5 and harvested embryos at E18.5 or at 5 months of age. We found that E18.5 *Atoh1* ^*CreERT2/+*^; *ROSA* ^*sT*^ mice had ~50% more Merkel cells than littermate controls, while 5 month-old *Atoh1* ^*CreERT2/+*^; *ROSA* ^*sT*^ mice had ~30% fewer Merkel cells than their littermate controls (Fig 6F–6J). No tumors were found at either age. These data are consistent with sT expression promoting mitosis in actively dividing cell populations, and demonstrate that touch dome Merkel cell numbers are regulated even in the face of this increased division. As in adult animals, MCV sT expression does not transform these cells.

To determine if combined sT expression and loss of p53 would promote Merkel cell proliferation, we administered TMX to E14.5 *Atoh1* ^*CreERT2/+*^; *ROSA* ^*sT*^; *p53* ^*flox/flox*^ mice and harvested skin at P28. We found no increases in Merkel cell numbers and no evidence of tumorigenesis (Fig 6K–6M). Unlike in sT-induced liver and spleen neoplasia, these data suggest that sT expression coupled with *p53* deletion is insufficient to promote sustained Merkel cell division or tumorigenesis.

## Discussion

MCV sT transforms immortalized rodent fibroblasts such as Rat1 and NIH3T3 [14] and viral sT expression is required for growth of MCV-positive MCC cancer cell lines [15, 16]. Our current data suggests that MCV expression in the context of p53 loss promotes fully neoplastic lesions in mice. We find that MCV sT induces embryonic Merkel cell proliferation but is insufficient to fully recapitulate Merkel cell carcinoma regardless of p53 status.

Recently, Verhaegen et al demonstrated that epithelium-specific sT expression using a K5 promoter in C57BL/6 mice induced hyperplastic lesions resembling squamous cell carcinoma *in situ* [18]. This proliferative effect was mediated through the MCV sT LSD domain, which has been found to target multiple E3 ligase tumor suppressor proteins [12, 13]. In contrast, we found that ubiquitous postnatal induction of MCV sT expression was uniformly lethal to mice after high-dose TMX injection. Our transgenic mouse model employs a codon-optimized MCV sT cDNA, which results in higher protein expression than the natural sT cDNA sequence (data not shown), and cytotoxic effects from increased levels of MCV sT expression may account for this difference. Ear, skin, muscle and brain tissues maintained sT expression long after low-dose TMX injection, suggesting that mice can tolerate chimeric expression of MCV sT.

Spurgeon et al. also reported that when tumor-derived genomic T antigen (both sT and MCC-derived truncated LT) is expressed under a constitutive K14 promoter in FVB/N background mice (*K14-MCPyV168*), two-thirds of mice are viable, whereas the rest of the mice died or were euthanized at early age due to malnourishment [17]. We obtained similar results in our model. How MCV sT exerts its lethal effects in mouse cells remains unknown. In human MCC, however, MCV LT may exert a modulatory activity that alleviates MCV sT lethality.

When lower doses of TMX were used to induce sT expression, nearly 70% of *Ubc* ^*CreERT2*^; *ROSA* ^*sT*^ mice survived over 10 days. These mice exhibit a hyperproliferative ear skin phenotype, similar to skin hyperplasias described in K5 and K14 tissue specific expression transgenic mouse models [17, 18]. MCV sT also increased production of Merkel cells in embryonic *Atoh1* ^*CreERT2/+*^; *ROSA* ^*sT*^ mice. Collectively, these results indicate that sT is a strong growth accelerator in mouse skin tissue. Despite sT’s proliferative effect, none of the C57BL/6 mice expressing MCV sT developed fully transformed neoplasia.

Simultaneous induction of MCV sT expression and p53 knockout in C57BL/6 mice, however, leads to the development of poorly-differentiated neoplasia in the spleen and liver, suggesting the importance of the p53 tumor suppressor pathway in sT-induced tumorigenesis. In line with these results, sT efficiently induces cell transformation only in the MEFs with p53 deletion. Further, tissues harvested at autopsy from multiple high-dose TMX-treated mice revealed widespread positivity for apoptosis in intestines, lung, liver and spleen. One explanation for these findings is that sT driven mitogenesis may induce mitotic catastrophe and p53 activation. p53 absence therefore may be protective to cells that would otherwise be eliminated through a p53-dependent apoptotic response.

This is also consistent with epithelium-specific MCV T antigen transgenic mouse models, which show activation of the double strand DNA damage marker γH2AX in hyperproliferative skin epithelium [17, 18]. Oncogene-induced hyperproliferation generally promotes the DNA damage response (DDR), leading to p53 activation and consequent growth suppression, senescence or apoptosis [29]. Ablation of this anti-oncogenic barrier in early carcinogenesis is critical for malignant tumor formation [30]. Unlike SV40 LT, however, neither full length MCV LT nor MCC-derived truncated LT binds to p53 [9, 10], even though a recent study reports that MCV LT inhibits transcriptional activity of p53 [10]. Because mutations in the p53 gene are uncommon in MCC tumors [31], upstream or downstream signaling events in the p53 pathway may be inhibited in MCV-related MCC tumors. sT transformation of immortalized rodent fibroblast may also be enhanced by sT induced defects in downstream signaling in the p53 pathway, since the p21 gene promoter in Rat1 cells, which can be efficiently transformed by MCV sT alone, is hypermethylated and does not respond to DNA damage-induced p53 activation [32]. Although MCV oncogene expression and immune suppression are critical for the genesis of most MCC tumors [33], no obvious or consistent complementing cellular mutations have been found for this cancer [34].

In our study, transgenic sT expression was sufficient to promote increased proliferation of embryonic *Atoh1*-expressing Merkel cell precursors. However, this effect was short-lived and did not occur in adult mice, a finding consistent with the majority of adult Merkel cells being post-mitotic or terminally differentiated. Concomitant deletion of *p53* in *Atoh1* ^*CreERT2/+*^; *ROSA* ^*sT*^; *p53* ^*flox/flox*^ mice also failed to promote sustained proliferation or tumorigenesis. It is possible that LT targeting of RB and RB-related proteins, which has also been shown to be required for the maintenance of terminal cell differentiation state [35, 36] as well as LT-induced cell proliferation and MCC cell line survival [9, 11], is a necessary component for MCC carcinogenesis. Whether other cellular pathways must also be abrogated to initiate Merkel cell transformation *in vivo* is unknown.

Alternatively, it is possible that Merkel cell carcinoma does not arise from Merkel cells, but rather from another skin lineage. Ectopic expression of *Atoh1* in the skin is sufficient to activate the expression of several Merkel cell markers [37]. If MCV induces cell proliferation of another skin lineage, and *Atoh1* expression is activated either by the virus itself or by complementing mutations, the resulting tumor cells would express several Merkel cell markers and would appear to have arisen from the Merkel cell lineage. Further experiments are necessary to test this hypothesis.

Our results suggest that sT exerts its effects in a variety of organs and tissues. This is evidenced by the hyperproliferation seen *in vivo* not only in the epidermis but also in the dermal compartment of mouse ears. In support of the latter, *in vitro* induction of sT in isolated MEFs from *Ubc* ^*CreERT2*^; *ROSA* ^*sT*^ mice showed increased growth compared to *ROSA* ^*sT*^ controls. In the p53 null setting, sT-induced neoplasia developed at various internal sites. At least in the kidney, microscopy coupled with immunohistochemical localization indicates that sT-expressing epithelial cells (CK8 positive) lining renal tubules undergo proliferative changes ranging from in situ hyperplasia to invasive carcinoma. For the high-grade tumors found in spleen and liver, the degree of anaplasia exhibited by the tumor cells preclude morphologic classification of their cell of origin, and immunohistochemical analysis showed co-expression of disparate lineage markers. ASMA was most consistently expressed in the splenic and hepatic tumors, however, K14 was also focally expressed. NCAM a neural cell marker was also faintly expressed. In contrast to proliferative lesions in the kidney, tumors in the spleen and liver were CK8 negative. Hematopoietic makers were negative in tumors from all sites. It is not uncommon for poorly differentiated, highly anaplastic tumors to either lose phenotypic markers indicative of origin or gain anomalous expression of markers due to dysregulation of transcriptional factors. Our study does not identify the cell of origin in human Merkel cell carcinoma, but clearly supports the transforming properties of MCV sT in a variety of cell lineages and describes a robust mouse model for malignant transformation by MCV sT.

## Materials and Methods

### Ethics Statement

All mice were maintained in accordance with Institutional Animal Care and Use Committee (IACUC) guidelines at the University of Pittsburgh, and mice experiments were performed with approval from the Animal Ethics Committee of the University of Pittsburgh (IACUC breeding protocol #12010072 and IACUC experimental protocols #12020149 and # 13082143). TMX injections, mice monitoring, and euthanasia were carried out under conditions to minimize pain and suffering in compliance with IACUC guidelines at the University of Pittsburgh Hillman Cancer Center and the Children’s Hospital of Pittsburgh of the University of Pittsburgh Medical Center.

### Rosa26 Targeting Vector Construction

Codon-optimized MCV small T (sTco) cDNA was used for the initial ROSA26 targeting vector construction [14]. The sTco cDNA was isolated from the pcDNA6 vector using HincII and XhoI restriction endonuclease digestion, which drops out a 571bp fragment. This fragment was first cloned into G194 vector (Genoway) digested with SmaI and XhoI. The resulting plasmid was then digested with XhoI and SgrAI to isolate a 585 bp fragment including the sTco cDNA and hGH polyA, which was cloned into ARC1-pA vector (Genoway) using PshAI and SmaI restriction sites. The resulting 15648 bp plasmid is called PAM1-HR plasmid and contains necessary homology regions and the *loxP* flanked STOP/neomycin cassette (Fig 1A). Genoway validated the final targeting vector by restriction analysis as well as sequencing of coding exons. In addition, sequences of junctions between homology arms and selection cassette as well as junctions between homology arms and plasmid backbone were confirmed.

### Homologous Recombination in ES Cells

40 μg of linearized PAM1-HR targeting plasmid DNA were electroporated into 5x10^6^ C57BL/6 ES cells (260 Volt, 500 μF). ES cells were subjected to 200 μg/mL G418 selection 48 hours after electroporation. Electroporation was performed 3 times and a total of 1577 clones were isolated and amplified on gelatin in 96 well plates. The isolated clones were screened for 5’ and 3’ homologous recombination events by PCR and Southern blotting. The correct sTco sequence was confirmed in 13 out of 15 ES cell clones positive for recombination. 7 ES cell clones were injected into C57BL/6J-Tyrc-2J/J (C57BL/6 albino strain; white fur color) blastocysts, which were re-implanted into OF1 pseudo-pregnant females. The resulting chimeric offspring were screened for the contribution of recombinant ES cells according to their coat color (percentage of light dark patches was determined). Only one clone from two highly chimeric males with chimeric rate above 50% was used for breeding to C57BL/6 wildtype females for germ line transmission. F1 animals were genotyped by PCR using the Phusion high fidelity polymerase kit (NEB). The F1 generation produced 1 heterozygous male and 2 heterozygous females, which were bred to each other in order to obtain homozygous animals.

### Cross to UbcCreERT2 and AtohCreERT2/+ Mice

To conditionally induce MCV sT expression by cre-loxP recombination in mice, Cre-ERT2 transgenic mice with C57BL/6;129S background (B6;129S-Tg(*Ubc* ^*CreERT2 -/+*^)1Ejb/J, JAX mice stock number 007001) or *Atoh1* ^*CreERT2*^ transgenic mouse [25] were crossed with MCV sT homozygous transgenic mice (*ROSA* ^*sT/sT*^) to generate *Ubc* ^CreERT2 -/+^; *ROSA* ^*sT/+*^ or *Atoh1Cre* ^*ERT2/+*^ *; ROSA* ^*sT/+*^ C57BL/6 mice. All offspring are heterozygous for the sT transgene (as per Mendelian genetics), and were genotyped only for the *UBC* ^*CreERT2*^ and *Atoh1* ^*CreERT2*^ transgenes. The Cre-ERT2 fusion protein consists of Cre recombinase fused to a triple mutant form of the human estrogen receptor harboring G400V/M543A/L544A triple mutations of the human estrogen receptor ligand-binding domain that does not bind its natural ligand. CreERT2 expression is driven under human Ubiquitin C (Ubc) constitutive promoter (*Ubc* ^*CreERT2*^) or Atoh1 promoter (*Atoh1* ^*CreERT2/+*^) promoter in mice, and thus TMX injection (0.2 mg/g or 0.02 mg/g for *Ubc* ^*CreERT2*^ and 0.25 mg/g for *Atoh1* ^*CreERT2/+*^) induces MCV sT protein expression in most tissues or Atoh1-positive cells including Merkel cells. In *Atoh1* ^*CreERT2/+*^ mice, the Atoh1 locus is targeted by a vector replacing its ORF with *Cre-ERT2* and neomycin phosphotransferase gene [25]. *ROSA* ^*sT/+*^ mice and *Ubc* ^CreERT2 -/+^ mice were used as controls and did not induce sT expression after TMX injection. Mice were monitored daily after TMX injection and euthanized by 100% carbon dioxide inhalation upon reaching an end-point defined as a constant weight loss reaching 20% of pre-TMX treatment weight, and/or clinical signs consistent with neurological involvement including ataxia, tremors and behavioral changes. No analgesics or anesthetics were used in the course of the experiment.

To induce p53 knockout and sT expression by TMX treatment, *ROSA* ^*sT/sT*^ C57BL/6 mice were crossed to B6.129P2-Trp53tm1^Brn^ (JAX number 008462) that harbors p53 gene exons 2–10 flanked by *loxP* sites to generate *ROSA* ^*sT/+*^ *; p53* ^*flox/flox*^ C57BL/6. *Ubc* ^*CreERT2-/+*^ C57BL/6 mice were also crossed with p53^flox/flox^ C57BL/6 mice to generate *Ubc* ^*CreERT2-/+*^ *; p53* ^*flox/flox*^ C57BL/6 mice. Finally *Ubc* ^*CreERT2-/+*^ *; p53* ^*flox/flox*^ C57BL/6 mice were crossed to *ROSA* ^*sT/sT*^ *; p53* ^*flox/flox*^ C57BL/6 mice to obtain *Ubc* ^*CreERT2-/+*^; *ROSA* ^*sT/+*^; *p53* ^*flox/flox*^ from approximately half of the littermate. *Atoh1* ^*CreERT2/+*^ [25] mice were mated to *ROSA* ^*ST/ST*^ mice to generate *Atoh1* ^*+/+*^ *; ROSA* ^*ST/+*^ and *Atoh1* ^*CreERT2/+*^; *ROSA* ^*ST/+*^ mice in the same litters. *ROSA* ^*sT/+*^ mice are indicated as *ROSA* ^*sT*^ in the text for simplification. Tamoxifen (0.25 mg/g) was administered by oral gavage to pregnant dams at E14.5 or adult mice at the specified ages. For embryonic ages, the plug date was designated as E0.5.

### Tissue Processing

Tissues from *Ubc* ^*CreERT2*^ mice studies were either quickly frozen or fixed in 10% buffered formalin (Sigma) for immunohistochemistry. *Atoh1* ^*+/+*^; *ROSA* ^*ST*^ and *Atoh1* ^*CreERT2/+*^; *ROSA* ^*ST*^ mice were euthanized by cervical dislocation and back skin dissected into cold PBS. Embryos were dissected from pregnant dams and decapitated before tissue dissection. Skin processed for whole-mount immunostaining was fixed in 4% PFA for 30 min (adult tissue or dissected embryonic skin) then washed in PBS. Some adult skin was embedded in OCT (Thermo Fisher Scientific), cryosectioned at 20μt 20ectione and counterstained with hematoxylin and eosin (Sigma-Aldrich).

### Genotyping

Genomic DNA was extracted by the NaOH-Tris method. Briefly, 100 μL 50 mM NaOH pH 13 was added to tubes containing ear snips and boiled at 95°C for 15 min. To neutralize the solution, 5 μL of 1.0 M Tris, pH 8.0 was added to the tube. Genomic DNA extracted into the supernatant was used for PCR. The genotyping PCR reaction for *MCV sT* contains 2 sets of primers to amplify the endogenous ROSA26 locus as well as the knock-in allele, which allows for the identification of wildtype, heterozygous or homozygous genotypes in one PCR run. Primers used to amplify the endogenous ROSA26 locus are forward primer #4719 (11053-11071(F)) and reverse primer #4723 (sTco_C-term.F.2). Primers used to amplify the knock-in allele are forward primer #4656 (027ROSA-PAM1) and reverse primer #4657 (028ROSA-PAM1). The PCR primer sets to detect *Cre-ERT2* are #4666 (genericCre.F) and #4667 (GenericCre.R). Those to detect p53 knock-in allele are #4931 (Trp53tm1Brn.mouse.F) and #4932 (Trp53tm1Brn.mouse.R). The PCR reaction was performed with an annealing temperature of 58°C for 30 cycles. Primer sequences and representative sT genotyping results are shown in S7 Fig. The expected product sizes are ~300 bp for the endogenous Rosa26 locus amplification and ~650 bp for the MCV sT knock-in allele amplification. Genotyping of offspring from het x het crosses reveals an approximate adherence to Mendelian genetics.

### Immunoblotting and Antibodies

Frozen mouse tissues were placed in separate tubes that were filled with ~1 mL of lysis buffer (50 mM Tris-HCl pH7.4, 0.15 M NaCl, 1% Triton X-100, 0.5% deoxycholate, 0.1% SDS, 2 mM Na_3_VO_4_ and 2 mM NaF) containing protease inhibitors (Complete, Roche). Tissues were minced in a tube by a surgical scissor on ice, transferred to FastPrep-24 lysing matrix tubes (MP Biomedicals), and homogenized by FastPrep tissue lyser (FP120/Bio101, Thermo Savant). Lysates were clarified by centrifugation at 13.2 x 10^3^ rpm for 10 min at 4°C, and supernatant was collected as soluble protein fraction. Lysates were then resolved by 12% SDS-PAGE and transferred to nitrocellulose. Membranes were blocked with 5% milk in 1X TBS and incubated with primary antibodies overnight at 4°C. Blots were subsequently incubated with IRDye-labeled anti-rabbit or anti-mouse secondary antibodies and analyzed on the Odyssey infrared scanner (LI-COR Biosciences). The previously described CM8E6 antibody [22] was used to detect MCV sT. c-Myc protein was detected by rabbit polyclonal antibody c-Myc (C-19) (Santa Cruz). A mouse monoclonal antibody to Hsp70 (Santa Cruz) was used to detect Hsp/Hsc70 as an internal control.

### Isolation of Mouse Embryonic Fibroblast Cells

Embryos (ED 13) from *Ubc* ^*CreERT2*^ *; ROSA* ^*sT*^ (n = 8), *Ubc* ^*CreERT2*^ *; ROSA* ^*sT*^ *; p53* ^*flox/flox*^ (n = 2) as well as *Ubc* ^*CreERT2*^ *; p53* ^*flox/flox*^; (n = 1) and *ROSA* ^*sT*^ (n = 2) were minced and plated individually in Dulbecco's modified Eagle's medium supplemented with 10% fetal calf serum. Most minced embryos attached to the plate by day 3 after plating. Mouse embryonic fibroblast cells (MEF) proliferated from minced embryos, were trypsinized and seeded onto culture dishes by day 10 after plating. Cells were trypsinized and seeded into new dishes as first passage MEF cells. To activate CreERT2, MEF cells were treated with 4OHTMX for 7 days.

### Immunohistochemistry

Mouse tissues were fixed with 10% neutral buffered formalin, embedded in paraffin, and 5μm sections were cut onto glass slides. Immunohistochemistry was performed with primary antibodies to MCV sT (CM5E1, 1:1,000) [14], α-smooth muscle actin (Biocare, CME305A.B, 1:300), CD45 (Millipore, 05–1416,1:300), Keratin14 (Biolegend, PRB-155P, 1:1000), Vimentin (Santa-cruz, sc-73258,1:300), CK8 (Developmental Studies Hybridoma Bank (DSHB), TROMA-I, 1:20), NCAM (DSHB, AG1, 1:50), and CD68 (Biocarta, CM033B,1:300). Anti-mouse IgG block are used for mouse primary antibodies, for primary antibodies produced in rat, a protein blocker (DAKO) was used. MCV sT was immunostained as described previously [14]. For remaining protein targets, 10 mM sodium citrate pH 6.0 buffer was used for heat-antigen retrieval (Digital Decloaking Chamber, Biocare). Primary antibody was reacted with biotinylated species-specific second antibodies, followed by the avidin-horse radish peroxidase (HRP) conjugation reaction. HRP activity was visualized using 3-amino-9-ethylcarbazole (AEC) or 3,3’-diaminobenzidine (DAB). Images were captured with an Olympus AX70 microscope using the Q-Capture Pro7 Program.

### Immunofluorescence

For immunofluorescence, body skin was cut into 2mm x 2mm pieces, washed with PBS containing 0.3% Triton X-100 (0.3% PBST) every 30 minutes for 5–8 h, then incubated with rat anti-keratin 8 (DSHB) diluted 1:20 in 0.3% PBST/5% goat serum/20% DMSO at room temperature for 3–5 days. Samples were then washed with 0.3% PBST every 30 minutes for 5–8 h and incubated in Cy3-conjugated donkey anti-rat secondary (Jackson ImmunoResearch Laboratories) diluted 1:250 in 0.3% PBST/5% goat serum/20% DMSO at room temperature for 2–4 days. Tissues were washed with 0.3% PBST every 30 minutes for 5–8 h then mounted on slides with Prolong Gold (Invitrogen).

### Cell Counts

Numbers of K8+ cells/touch dome were determined from wholemount, K8-immunostained skin pieces at least 1 cm^2^ in size. All K8+ cells in at least 20 touch domes from 2–6 mice per genotype were counted; no correction factors were applied.

### Statistical Analysis

To evaluate statistical significance between two survival curves, the log-rank (Mantel-Cox) test was performed (PRISM). Statistical comparisons between genotypes in soft agar colonies and Merkel cell numbers were done using two-tailed t-tests (Excel).

## Supporting Information

S1 Fig Dose-dependent induction of sT protein expression by TMX and induction of cellular apoptosis by sT in mice. (A) Quantification of sT protein expression in multiple tissues from *Ubc* ^*CreERT2*^ *; ROSA* ^*sT*^ mice surviving <10 days shown in Fig 1C. sT protein expression was normalized by hsp70 loading control, and relative abundance to equally-loaded control (sT transfected 293) was determined. Quantification was performed by Li-COR infrared immunoblot. (B) Induction of TUNEL positive cells in *Ubc* ^*CreERT2*^ *; ROSA* ^*sT*^ mice that were sacrificed at day 3 after multiple high dose TMX treatment (Injected with 0.2 mg/g TMX each day for 3 days).
(PDF)

S2 Fig MCV sT induces hyperproliferation in MEF cells. (A) MEF cells treated with 500 nM 4OHTMX for 7 days were subjected to sT immunoblots by CM8E6 antibody and c-Myc immunoblots. αTubulin was used as a loading control. Littermate 4 and 6 show leaky sT expression in the absence of 4OHTMX treatment. (B) MCV sT-induced growth acceleration was observed in multiple MEFs except littermate 4, which showed highest leaky expression. Proliferation of MEF cells treated with or without 4-OHTMX was evaluated by Wst1 assays.
(PDF)

S3 Fig p53 ablation does not rescue MCV sT-induced lethality in mice. (A) MCV sT induction by high dose TMX treatment is lethal regardless of p53 status, and the lethality is TMX dose-dependent. Kaplan-Meier curve from high dose (0.2 mg/g), low dose (0.02 mg/g) and 1/2 low dose (0.01 mg/g) TMX-injected *Ubc* ^*CreERT2*^ *; ROSA* ^*sT*^ *; p53* ^*flox/flox*^ mice. (B) Multi-tissue MCV sT protein expression in *Ubc* ^*CreERT2*^ *; ROSA* ^*sT*^ *; p53* ^*flox/flox*^ mice that died at 8 days after low dose TMX injection. MCV sT protein expression was detected by CM8E6 immunoblotting. MCV sT or empty vector transfected 293 cell lysates were used as a positive and a negative control, respectively. Equal amounts of sT-transfected 293 cells lysates were loaded for normalization across different blots.
(PDF)

S4 Fig Hyperplastic phenotype in ear skin from *Ubc* ^*CreERT2*^ *; ROSA* ^*sT*^ *; p53* ^*flox/flox*^ mice. (A) Representative histology of thickened ear from *Ubc* ^*CreERT2*^ *; ROSA* ^*sT*^ *; p53* ^*flox/flox*^ mice (left) and a control ear from *ROSA* ^*sT*^ *; p53* ^*flox/flox*^ mice. (B) A representative H&E-stained section of ear skin from *Ubc* ^*CreERT2*^ *; ROSA* ^*sT*^ *; p53* ^*flox/flox*^ mice.
(PDF)

S5 Fig MCV sT protein expression in *Ubc* ^*CreERT2*^ *; ROSA* ^*sT*^ *; p53* ^*flox/flox*^ mice that survived over 60 days post TMX injection. sT expression was maintained in 80% of ear skin tissues (4/5). 75% (3/4) of mice bearing spleen tumors showed sT protein. Except for p53.7F, sT expression was not detectable in liver tumors because tumors were not macroscopically visible and could not be excised. MCV sT protein was detected by immunoblot with CM8E6 antibody. Hsp/Hsc70 expression was used as a loading control.
(PDF)

S6 Fig sT induced tumors do not originate from macrophages or lymphocytes. sT-induced liver tumors are negative for CD68 (macrophage marker) and CD45 (lymphocyte marker). sT-induced liver tumors from p53.7F and control tissues were subjected to immunohistochemical staining with CD68 and CD45. Normal spleen (*Ubc* ^*CreERT2*^ for CD68 and *Ubc* ^*CreERT2*^ *; p53* ^*flox/flox*^ for CD45) was used as a positive control for each marker staining.
(PDF)

S7 Fig Genotyping PCR primers. (A) PCR primers that were used to detect *ROSA* ^*sT*^ (sT knock-in allele), *ROSA* ^*WT*^ alleles, *Cre-ERT2*, *p53* ^*floxed*^ allele are shown. (B) Representative *ROSA* ^*sT*^ genotyping results for 10 littermates from *Ubc* ^*Cre -/+*^ and *ROSA* ^*sT+/+*^ mating. Red arrow indicates PCR products from *ROSA* ^*sT*^ (~650bp) while blue arrow from wild type *ROSA* ^*+*^ (~300 bp) allele.
(PDF)

## References

[1] Feng H, Shuda M, Chang Y, Moore PS. Clonal integration of a polyomavirus in human Merkel cell carcinoma. Science. 2008;319(5866):1096–100. 10.1126/science.1152586 18202256 PMC2740911

[2] DeCaprio JA, Garcea RL. A cornucopia of human polyomaviruses. Nature reviews Microbiology. 2013;11(4):264–76. 10.1038/nrmicro2992 23474680 PMC3928796

[3] Feng H, Taylor JL, Benos PV, Newton R, Waddell K, Lucas SB, et al Human transcriptome subtraction by using short sequence tags to search for tumor viruses in conjunctival carcinoma. Journal of virology. 2007;81(20):11332–40. 10.1128/JVI.00875-07 17686852 PMC2045575

[4] Borozan I, Watt SN, Ferretti V. Evaluation of alignment algorithms for discovery and identification of pathogens using RNA-Seq. PloS one. 2013;8(10):e76935 10.1371/journal.pone.0076935 24204709 PMC3813700

[5] Shuda M, Feng H, Kwun HJ, Rosen ST, Gjoerup O, Moore PS, et al T antigen mutations are a human tumor-specific signature for Merkel cell polyomavirus. Proc Natl Acad Sci U S A. 2008;105(42):16272–7. 10.1073/pnas.0806526105 18812503 PMC2551627

[6] Wendzicki JA, Moore PS, Chang Y. Large T and small T antigens of Merkel cell polyomavirus. Current opinion in virology. 2015;11:38–43. 10.1016/j.coviro.2015.01.009 25681708 PMC4456251

[7] Kassem A, Schopflin A, Diaz C, Weyers W, Stickeler E, Werner M, et al Frequent detection of Merkel cell polyomavirus in human Merkel cell carcinomas and identification of a unique deletion in the VP1 gene. Cancer research. 2008;68(13):5009–13. 10.1158/0008-5472.CAN-08-0949 . 18593898

[8] Schmitt M, Wieland U, Kreuter A, Pawlita M. C-terminal deletions of Merkel cell polyomavirus large T-antigen, a highly specific surrogate marker for virally induced malignancy. International journal of cancer Journal international du cancer. 2012;131(12):2863–8. 10.1002/ijc.27607 . 22674148

[9] Cheng J, Rozenblatt-Rosen O, Paulson KG, Nghiem P, DeCaprio JA. Merkel cell polyomavirus large T antigen has growth-promoting and inhibitory activities. Journal of virology. 2013;87(11):6118–26. 10.1128/JVI.00385-13 23514892 PMC3648111

[10] Borchert S, Czech-Sioli M, Neumann F, Schmidt C, Wimmer P, Dobner T, et al High-affinity Rb binding, p53 inhibition, subcellular localization, and transformation by wild-type or tumor-derived shortened Merkel cell polyomavirus large T antigens. Journal of virology. 2014;88(6):3144–60. 10.1128/JVI.02916-13 24371076 PMC3957953

[11] Houben R, Adam C, Baeurle A, Hesbacher S, Grimm J, Angermeyer S, et al An intact retinoblastoma protein-binding site in Merkel cell polyomavirus large T antigen is required for promoting growth of Merkel cell carcinoma cells. International journal of cancer Journal international du cancer. 2012;130(4):847–56. 10.1002/ijc.26076 . 21413015

[12] Kwun HJ, Shuda M, Feng H, Camacho CJ, Moore PS, Chang Y. Merkel cell polyomavirus small T antigen controls viral replication and oncoprotein expression by targeting the cellular ubiquitin ligase SCFFbw7. Cell Host Microbe. 2013;14(2):125–35. 10.1016/j.chom.2013.06.008 23954152 PMC3764649

[13] Shuda M, Velasquez C, Cheng E, Cordek DG, Kwun HJ, Chang Y, et al CDK1 substitutes for mTOR kinase to activate mitotic cap-dependent protein translation. Proceedings of the National Academy of Sciences of the United States of America. 2015 10.1073/pnas.1505787112 . 25883264 PMC4434708

[14] Shuda M, Kwun HJ, Feng H, Chang Y, Moore PS. Human Merkel cell polyomavirus small T antigen is an oncoprotein targeting the 4E-BP1 translation regulator. J Clin Invest. 2011;121(9):3623–34. 10.1172/JCI46323 21841310 PMC3163959

[15] Houben R, Shuda M, Weinkam R, Schrama D, Feng H, Chang Y, et al Merkel cell polyomavirus-infected Merkel cell carcinoma cells require expression of viral T antigens. J Virol. 2010;84(14):7064–72. 10.1128/JVI.02400-09 20444890 PMC2898224

[16] Shuda M, Chang Y, Moore PS. Merkel cell polyomavirus-positive Merkel cell carcinoma requires viral small T-antigen for cell proliferation. J Invest Dermatol. 2014;134(5):1479–81. 10.1038/jid.2013.483 24217011 PMC3989379

[17] Spurgeon ME, Cheng J, Bronson RT, Lambert PF, DeCaprio JA. Tumorigenic activity of Merkel cell polyomavirus T antigens expressed in the stratified epithelium of mice. Cancer research. 2015 10.1158/0008-5472.CAN-14-2425 . 25596282 PMC4359959

[18] Verhaegen ME, Mangelberger D, Harms PW, Vozheiko TD, Weick JW, Wilbert DM, et al Merkel Cell Polyomavirus Small T Antigen Is Oncogenic in Transgenic Mice. The Journal of investigative dermatology. 2014 10.1038/jid.2014.446 . 25313532 PMC4397111

[19] Blyth K, Terry A, O'Hara M, Baxter EW, Campbell M, Stewart M, et al Synergy between a human c-myc transgene and p53 null genotype in murine thymic lymphomas: contrasting effects of homozygous and heterozygous p53 loss. Oncogene. 1995;10(9):1717–23. . 7753548

[20] Donehower LA, Godley LA, Aldaz CM, Pyle R, Shi YP, Pinkel D, et al Deficiency of p53 accelerates mammary tumorigenesis in Wnt-1 transgenic mice and promotes chromosomal instability. Genes & development. 1995;9(7):882–95. . 7705663 10.1101/gad.9.7.882

[21] Symonds H, Krall L, Remington L, Saenz-Robles M, Lowe S, Jacks T, et al p53-dependent apoptosis suppresses tumor growth and progression in vivo. Cell. 1994;78(4):703–11. . 8069917 10.1016/0092-8674(94)90534-7

[22] Kwun HJ, Guastafierro A, Shuda M, Meinke G, Bohm A, Moore PS, et al The minimum replication origin of merkel cell polyomavirus has a unique large T-antigen loading architecture and requires small T-antigen expression for optimal replication. J Virol. 2009;83(23):12118–28. 10.1128/JVI.01336-09 19759150 PMC2786723

[23] Jonkers J, Meuwissen R, van der Gulden H, Peterse H, van der Valk M, Berns A. Synergistic tumor suppressor activity of BRCA2 and p53 in a conditional mouse model for breast cancer. Nature genetics. 2001;29(4):418–25. 10.1038/ng747 . 11694875

[24] Choi J, Curtis SJ, Roy DM, Flesken-Nikitin A, Nikitin AY. Local mesenchymal stem/progenitor cells are a preferential target for initiation of adult soft tissue sarcomas associated with p53 and Rb deficiency. The American journal of pathology. 2010;177(5):2645–58. 10.2353/ajpath.2010.100306 20864684 PMC2966819

[25] Fujiyama T, Yamada M, Terao M, Terashima T, Hioki H, Inoue YU, et al Inhibitory and excitatory subtypes of cochlear nucleus neurons are defined by distinct bHLH transcription factors, Ptf1a and Atoh1. Development. 2009;136(12):2049–58. 10.1242/dev.033480 . 19439493

[26] Bossuyt W, Kazanjian A, De Geest N, Van Kelst S, De Hertogh G, Geboes K, et al Atonal homolog 1 is a tumor suppressor gene. PLoS biology. 2009;7(2):e39 10.1371/journal.pbio.1000039 19243219 PMC2652388

[27] Maricich SM, Wellnitz SA, Nelson AM, Lesniak DR, Gerling GJ, Lumpkin EA, et al Merkel cells are essential for light-touch responses. Science. 2009;324(5934):1580–2. 10.1126/science.1172890 19541997 PMC2743005

[28] Wright MC, Reed-Geaghan EG, Bolock AM, Fujiyama T, Hoshino M, Maricich SM. Unipotent, Atoh1+ progenitors maintain the Merkel cell population in embryonic and adult mice. The Journal of cell biology. 2015;208(3):367–79. 10.1083/jcb.201407101 25624394 PMC4315254

[29] Halazonetis TD, Gorgoulis VG, Bartek J. An oncogene-induced DNA damage model for cancer development. Science. 2008;319(5868):1352–5. 10.1126/science.1140735 . 18323444

[30] Bartkova J, Horejsi Z, Koed K, Kramer A, Tort F, Zieger K, et al DNA damage response as a candidate anti-cancer barrier in early human tumorigenesis. Nature. 2005;434(7035):864–70. 10.1038/nature03482 . 15829956

[31] Schmid M, Janssen K, Dockhorn-Dworniczak B, Metze D, Zelger BW, Luger TA, et al p53 abnormalities are rare events in neuroendocrine (Merkel cell) carcinoma of the skin. An immunohistochemical and SSCP analysis. Virchows Archiv: an international journal of pathology. 1997;430(3):233–7. . 9099981 10.1007/BF01324807

[32] Allan LA, Duhig T, Read M, Fried M. The p21(WAF1/CIP1) promoter is methylated in Rat-1 cells: stable restoration of p53-dependent p21(WAF1/CIP1) expression after transfection of a genomic clone containing the p21(WAF1/CIP1) gene. Molecular and cellular biology. 2000;20(4):1291–8. 10648615 10.1128/mcb.20.4.1291-1298.2000 PMC85267

[33] Moore PS, Chang Y. Why do viruses cause cancer? Highlights of the first century of human tumour virology. Nature reviews Cancer. 2010;10(12):878–89. 10.1038/nrc2961 21102637 PMC3718018

[34] Rodig SJ, Cheng J, Wardzala J, DoRosario A, Scanlon JJ, Laga AC, et al Improved detection suggests all Merkel cell carcinomas harbor Merkel polyomavirus. The Journal of clinical investigation. 2012;122(12):4645–53. 10.1172/JCI64116 23114601 PMC3533549

[35] Schneider JW, Gu W, Zhu L, Mahdavi V, Nadal-Ginard B. Reversal of terminal differentiation mediated by p107 in Rb-/- muscle cells. Science. 1994;264(5164):1467–71. . 8197461 10.1126/science.8197461

[36] Khidr L, Chen PL. RB, the conductor that orchestrates life, death and differentiation. Oncogene. 2006;25(38):5210–9. 10.1038/sj.onc.1209612 . 16936739

[37] Ostrowski SM, Wright M.C., Bolock A., Geng X. and Maricich S.M. Ectopic Atoh1 expression drives Merkel cell production in embryonic, postnatal and adult epidermis. Development. 2015;Accepted. 10.1242/dev.123141 PMC4510865 26138479

