# Supplementary figures and images for: Merkel Cell Polyomavirus Small T Antigen Induces Cancer and Embryonic Merkel Cell Proliferation in a Transgenic Mouse Model
Source: PLoS One. 2015 Nov 6;10(11):e0142329. doi: 10.1371/journal.pone.0142329 (PMC4636375; doi:10.1371/journal.pone.0142329)

S1 Fig

(A)

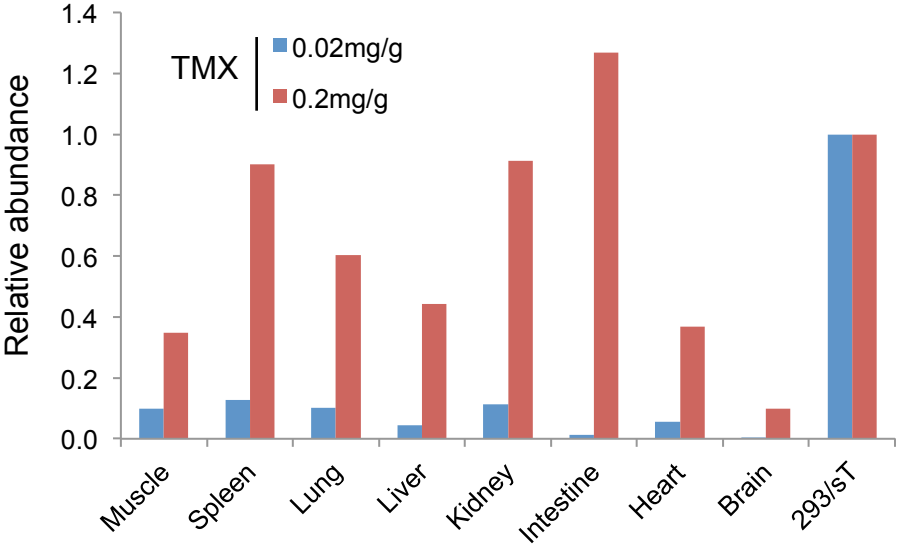

(B)

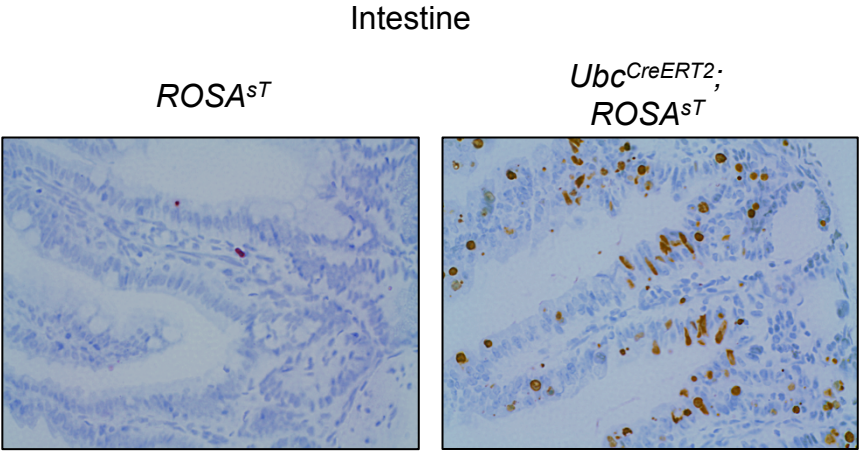

Supplement: S1 Fig — (A) Quantification of sT protein expression in multiple tissues from Ubc CreERT2 ; ROSA sT mice surviving <10 days shown in Fig 1C. sT protein expression was normalized by hsp70 loading control, and relative abundance to equally-loaded control (sT transfected 293) was determined. Quantification was performed by Li-COR infrared immunoblot. (B) Induction of TUNEL positive cells in Ubc CreERT2 ; ROSA sT mice that were sacrificed at day 3 after multiple high dose TMX treatment (Injected with 0.2 mg/g TMX each day for 3 days). (PDF) [file pone.0142329.s001.pdf]

**S2 Fig**

(A)

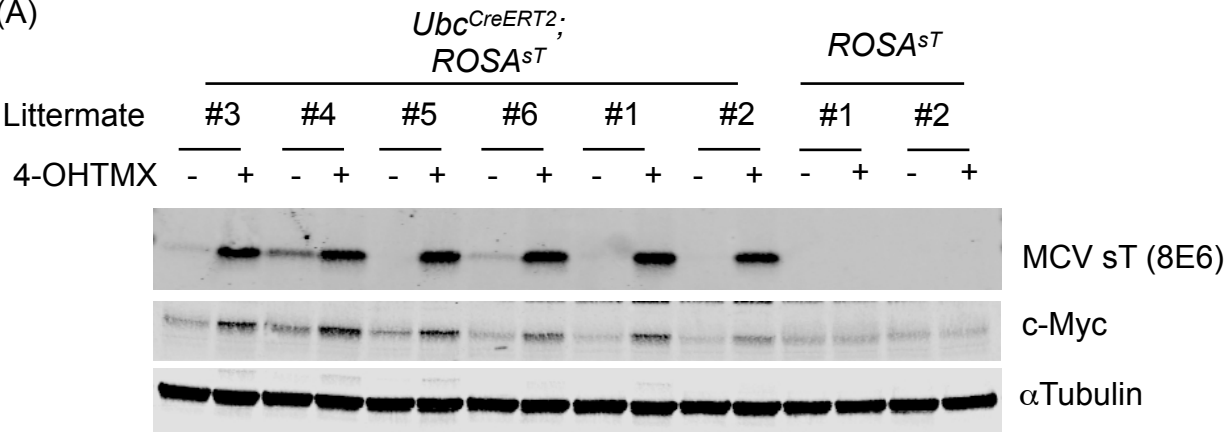

(B)

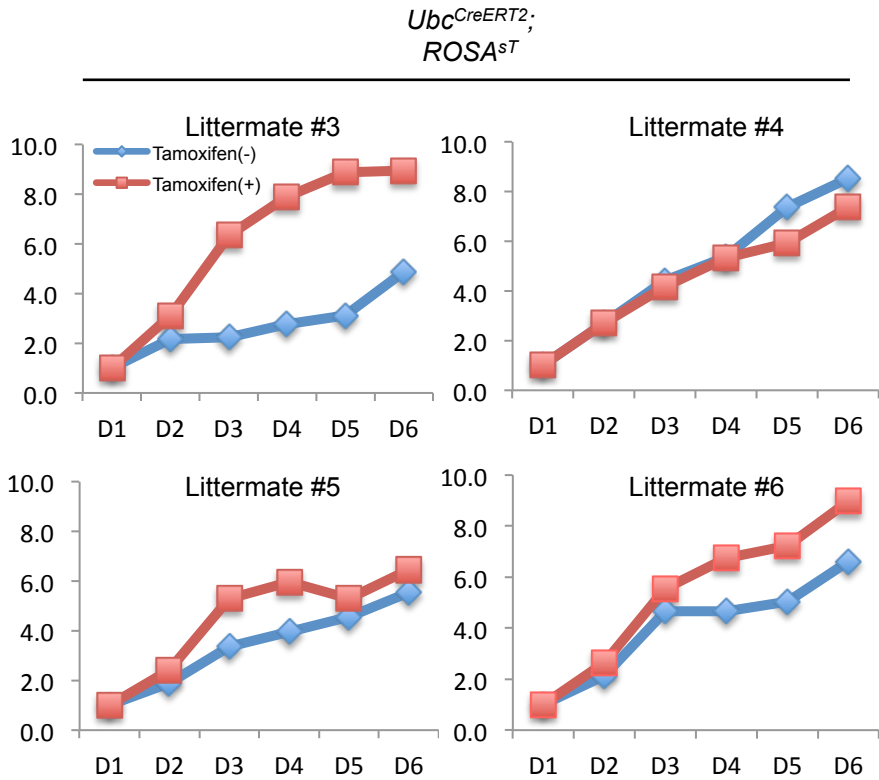

Supplement: S2 Fig — (A) MEF cells treated with 500 nM 4OHTMX for 7 days were subjected to sT immunoblots by CM8E6 antibody and c-Myc immunoblots. αTubulin was used as a loading control. Littermate 4 and 6 show leaky sT expression in the absence of 4OHTMX treatment. (B) MCV sT-induced growth acceleration was observed in multiple MEFs except littermate 4, which showed highest leaky expression. Proliferation of MEF cells treated with or without 4-OHTMX was evaluated by Wst1 assays. (PDF) [file pone.0142329.s002.pdf]

S3 Fig

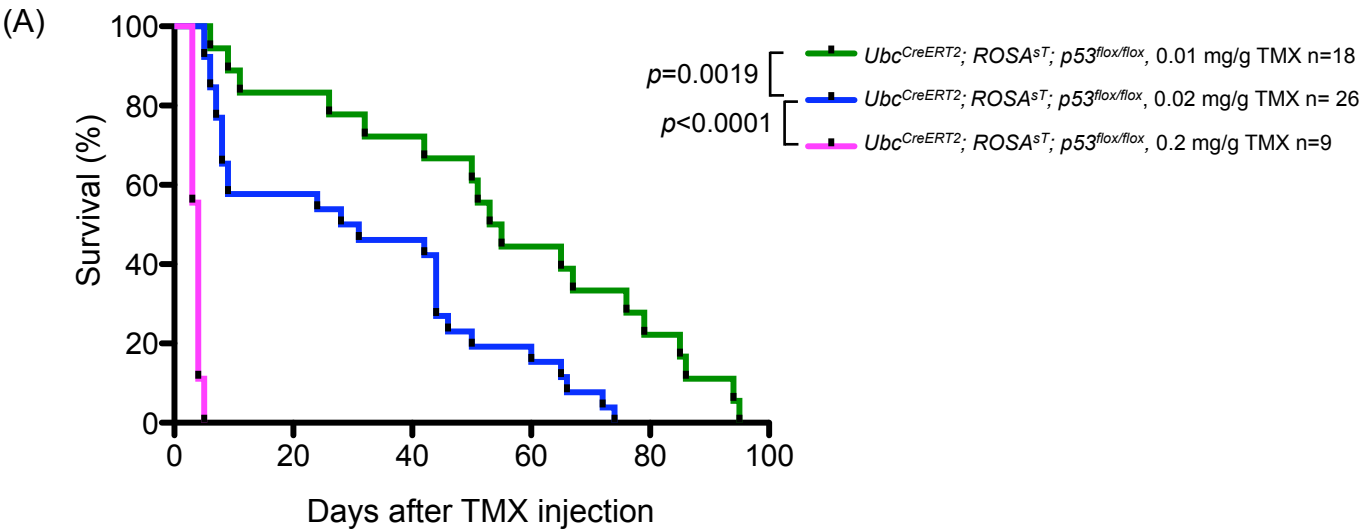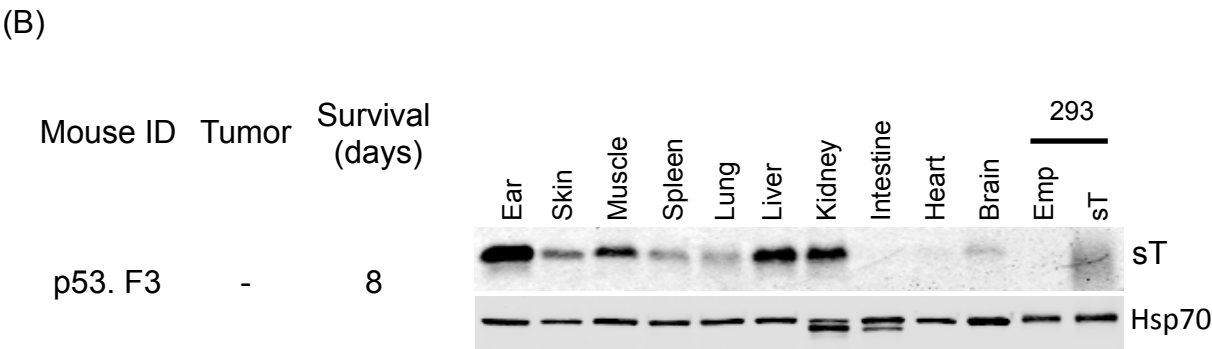

Supplement: S3 Fig — (A) MCV sT induction by high dose TMX treatment is lethal regardless of p53 status, and the lethality is TMX dose-dependent. Kaplan-Meier curve from high dose (0.2 mg/g), low dose (0.02 mg/g) and 1/2 low dose (0.01 mg/g) TMX-injected Ubc CreERT2 ; ROSA sT ; p53 flox/flox mice. (B) Multi-tissue MCV sT protein expression in Ubc CreERT2 ; ROSA sT ; p53 flox/flox mice that died at 8 days after low dose TMX injection. MCV sT protein expression was detected by CM8E6 immunoblotting. MCV sT or empty vector transfected 293 cell lysates were used as a positive and a negative control, respectively. Equal amounts of sT-transfected 293 cells lysates were loaded for normalization across different blots. (PDF) [file pone.0142329.s003.pdf]

## S4 Fig

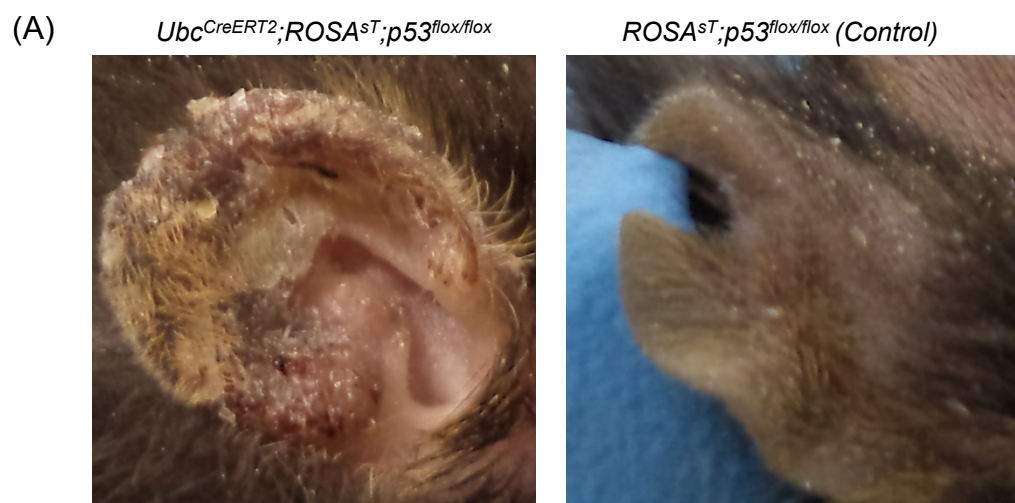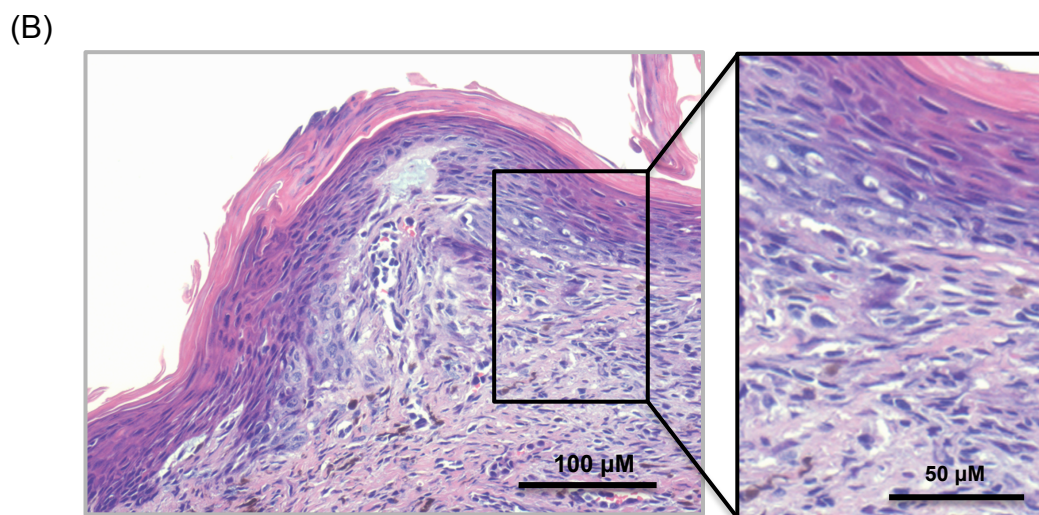

Supplement: S4 Fig — (A) Representative histology of thickened ear from Ubc CreERT2 ; ROSA sT ; p53 flox/flox mice (left) and a control ear from ROSA sT ; p53 flox/flox mice. (B) A representative H&E-stained section of ear skin from Ubc CreERT2 ; ROSA sT ; p53 flox/flox mice. (PDF) [file pone.0142329.s004.pdf]

S5 Fig

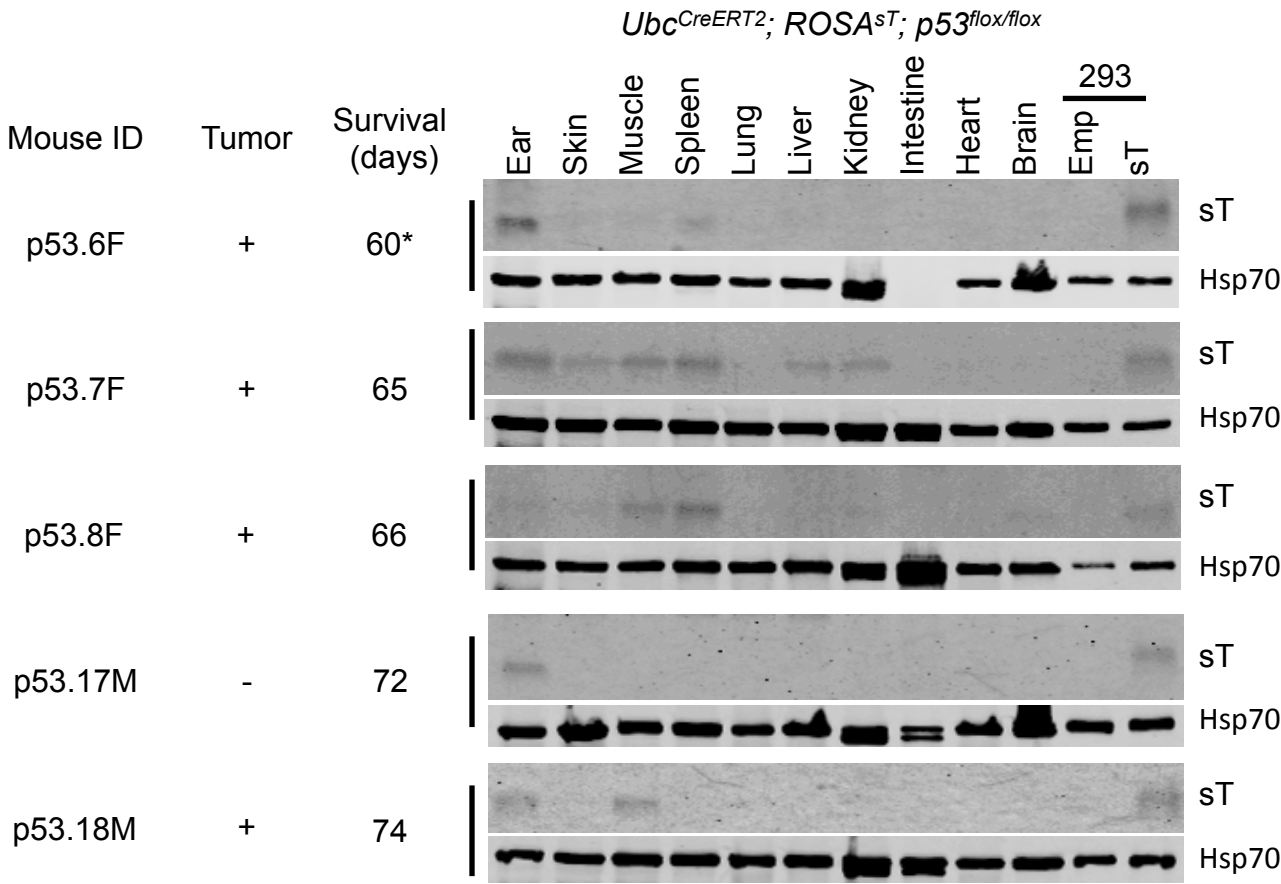

Supplement: S5 Fig — sT expression was maintained in 80% of ear skin tissues (4/5). 75% (3/4) of mice bearing spleen tumors showed sT protein. Except for p53.7F, sT expression was not detectable in liver tumors because tumors were not macroscopically visible and could not be excised. MCV sT protein was detected by immunoblot with CM8E6 antibody. Hsp/Hsc70 expression was used as a loading control. (PDF) [file pone.0142329.s005.pdf]

S6 Fig

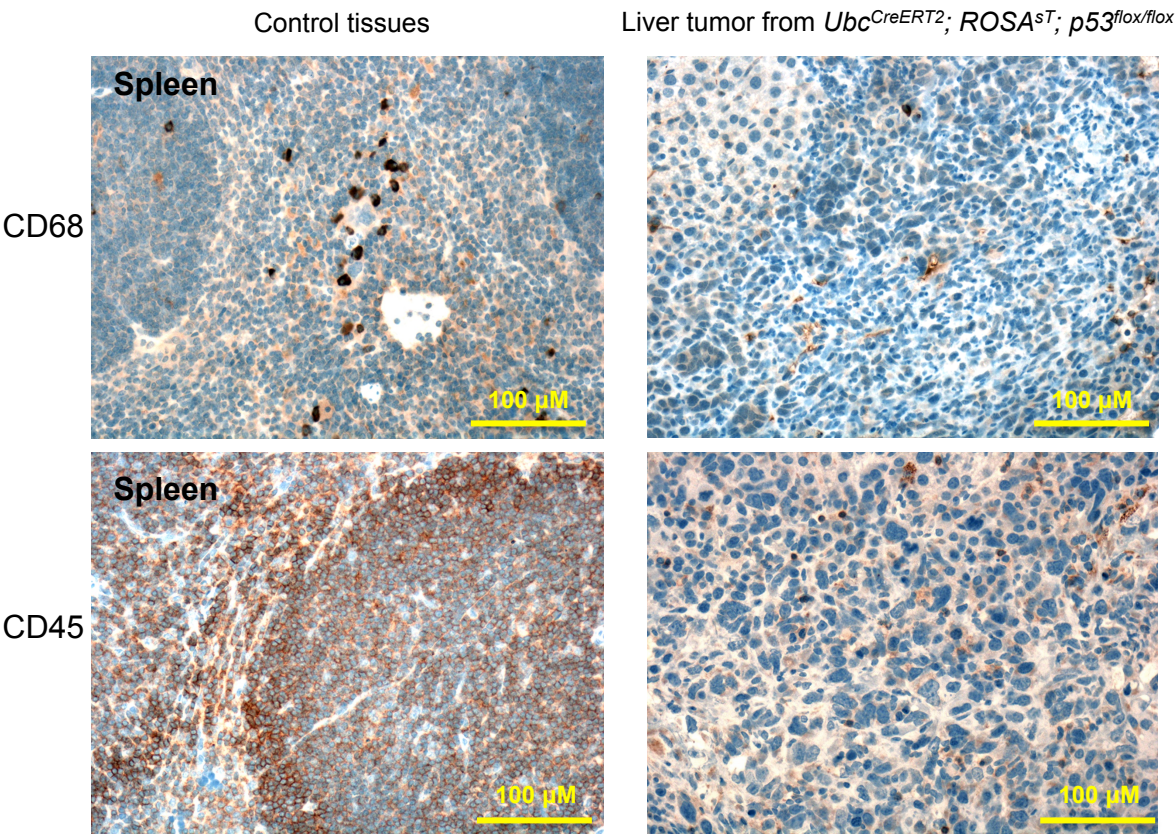

Supplement: S6 Fig — sT-induced liver tumors are negative for CD68 (macrophage marker) and CD45 (lymphocyte marker). sT-induced liver tumors from p53.7F and control tissues were subjected to immunohistochemical staining with CD68 and CD45. Normal spleen (Ubc CreERT2 for CD68 and Ubc CreERT2 ; p53 flox/flox for CD45) was used as a positive control for each marker staining. (PDF) [file pone.0142329.s006.pdf]
