# Supplementary material for: Merkel Cell Polyomavirus Small T Antigen Induces Cancer and Embryonic Merkel Cell Proliferation in a Transgenic Mouse Model
Source: PLoS One. 2015 Nov 6;10(11):e0142329. doi: 10.1371/journal.pone.0142329 (PMC4636375; doi:10.1371/journal.pone.0142329)

S7 Fig

(A)

Primer sequence for Transgenic mouse genotyping

Target	Primer #	Name	Primer sequence
ROSA^{sT} and wild type *ROSA*$^{+}$	#4719	11053-11071(F)	5'-TGG TTC ACG CCT GTA ATC -3'
	#4723	sTco_C-term.F.2	5'-CCG ACT ATT GCT TGC TTC AC -3'
	#4656	027Rosa-PAM1	5'-CAA TAC CTT TCT GGG AGT TCT CTG C -3'
	#4657	028Rosa-PAM1	5'-CTG CAT AAA ACC CCA GAT GAC TAC C -3'
Cre-ERT2	#4666	genericCre.F	5'-GCG GTC TGG CAG TAA AAA CTA TC -3'
	#4667	genericCre.R	5'-GTG AAA CAG CATT GCT GTC ACT T -3'
p53^{floxed}	#4931	Trp53tm1Brn.mouse.F	5'-GGT TAA ACC CAG CTT GAC CA -3'
	#4932	Trp53tm1Brn.mouse.R	5'-GGA GGC AGA GAC AGT TGG AG -3'

(B)

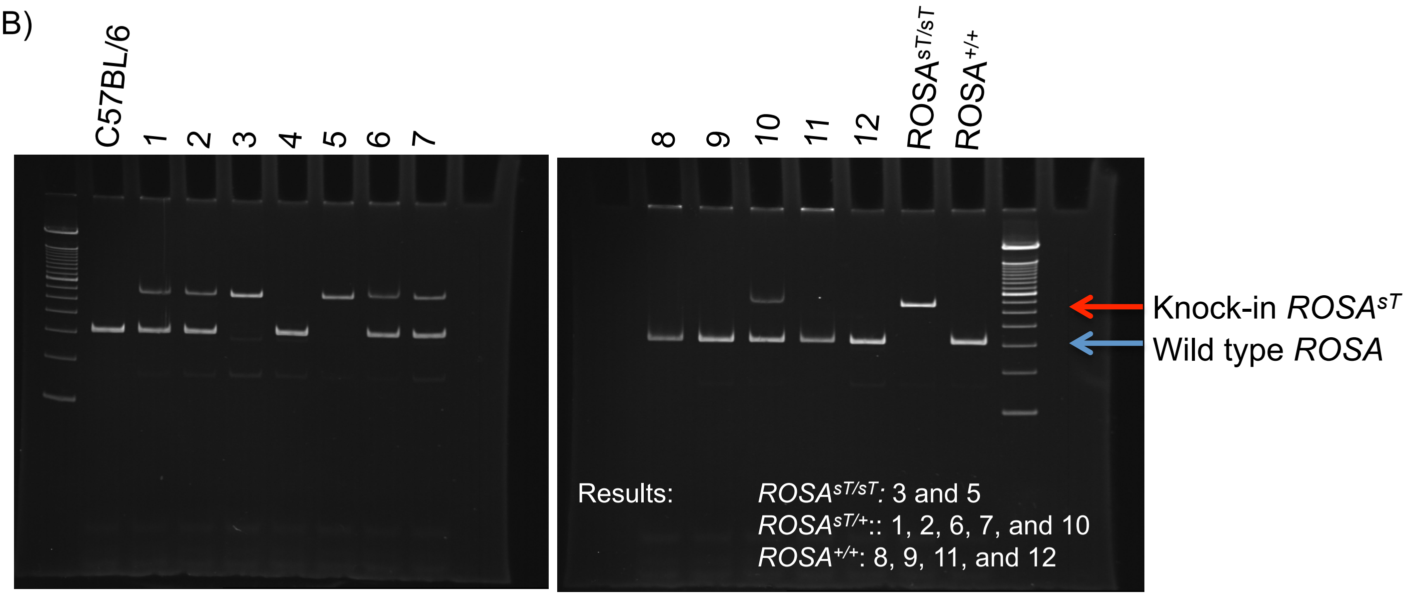

Supplement: S7 Fig — (A) PCR primers that were used to detect ROSA sT (sT knock-in allele), ROSA WT alleles, Cre-ERT2, p53 floxed allele are shown. (B) Representative ROSA sT genotyping results for 10 littermates from Ubc Cre -/+ and ROSA sT+/+ mating. Red arrow indicates PCR products from ROSA sT (~650bp) while blue arrow from wild type ROSA + (~300 bp) allele. (PDF) [file pone.0142329.s007.pdf]
